# Italian guidelines for the management of adult individuals with primary hypothyroidism outside pregnancy

**DOI:** 10.1007/s40618-025-02652-y

**Published:** 2025-07-23

**Authors:** Rinaldo Guglielmi, Caterina Mian, Roberto Novizio, Agostino Paoletta, Agnese Persichetti, Irene Samperi, Alessandro Scoppola, Marcello Bagnasco, Ernesto De Menis, Maria Luisa De Rimini, Marco Raffaelli, Maria Gabriella Rugiu, Claudio Andreoli, Marco Boniardi, Giulia Fiorentini, Pietro Locantore, Enrico Papini, Federica Presciuttini, Silvia Rizzati, Marsida Teliti, Doris Tina, Vincenzo Triggiani, Annibale Versari, Camilla Virili, Marina Vitillo, Michele Basile, Fabio Cruciani, Zuzana Mitrova, Rosella Saulle, Ilaria Valentini, Mirco Bartolomei, Giulia Bertino, Pietro Giorgio Calò, Federica D’Aurizio, Caterina Di Cosmo, Andrea Frasoldati, Massimo Marchetti, Rosa Elisa Miceli, Salvatore Monti, Alfredo Pontecorvi, Mario Rotondi, Roberto Attanasio

**Affiliations:** 1 Lifenet Health Group, Endocrinology, Regina Apostolorum Hospital, Albano Laziale, RM Italy; 2 Endocrinology, Department of Medicine DIMED, University of Padua, Padua, Italy; 3 Catholic University of the Sacred Heart, Rome, Italy; 4 Endocrinology Unit, AORN A. Cardarelli, Naples, Italy; 5 Endocrinology, ULSS6 Euganea, Padua, Italy; 6 Ministry of Interior - Department of Firefighters, Public Rescue and Civil Defense, Rome, Italy; 7 Department of Human Sciences and Promotion of the Quality of Life, San Raffaele Roma Open University, Rome, Italy; 8 Endocrinology, ASL Novara, Novara, Italy; 9 UOSD Endocrinologia, ASL Roma 1, Rome, Italy; 10 AIT President, Endocrinology Unit, Department of Internal Medicine and Medical Specialties (DiMI), University of Genoa, Genoa, Italy; 11 Internal Medicine 2, Treviso Hospital, Treviso, Italy; 12 AIMN President, Nuclear Medicine Unit, AORN Ospedali Dei Colli, Naples, Italy; 13 Endocrine and Metabolic Surgery Unit, Center for Research in Endocrine Gland and Obesity Surgery, Agostino Gemelli IRCCS University Polyclinic Foundation, Rome, Italy; 14 SOC di Otorinolaringoiatria, Ospedale di San Daniele e Tolmezzo, Azienda Sanitaria Universitaria Friuli Centrale, Udine, Italy; 15 Azienda ULSS 9 Scaligera, Verona, Italy; 16 Endocrine Surgery Unit, General Oncologic and Mini-invasive Surgery Department, ASST Grande Ospedale Metropolitano di Niguarda, Milan, Italy; 17 IRCCS San Raffaele, Rome, Italy; 18 Endocrinology Unit, Agostino Gemelli IRCCS University Polyclinic Foundation, Rome, Italy; 19 UOC Medicina – ASL, Rome, Italy; 20 UOC Medicina Interna, Ospedale S. Maria della Misericordia, Rovigo, Italy; 21 Unit of Endocrinology and Metabolism, Department of Internal Medicine and Therapeutics, ICS Maugeri Pavia, University of Pavia, Pavia, Italy; 22 UOC Malattie Endocrine e Diabetologia, PO Atri (TE), Milan, Italy; 23 Interdisciplinary Department of Medicine, Endocrinology, Geriatrics and Rare Diseases, University of Bari “Aldo Moro”, Bari, Italy; 24 Nuclear Medicine, Azienda Unità Sanitaria Locale–IRCCS di, Reggio Emilia, Reggio Emilia, Italy; 25 Endocrinology Unit, Department of Medico-Surgical Sciences and Biotechnologies, Sapienza University of Rome, Latina, Italy; 26 Clinical Pathology Unit, Laboratories Department, ASL Roma 1, Rome, Italy; 27 High School of Economy and Management of Health Systems, Catholic University of Sacred Heart, Rome, Italy; 28 Department of Epidemiology, Lazio Regional Health Service, ASL Roma 1, Rome, Italy; 29 Onco-Hematology Department, Nuclear Medicine Unit, University Hospital of Ferrara, Ferrara, Italy; 30 Department of Otorhinolaryngology, Head and Neck Surgery, University of Pavia, Foundation IRCCS Policlinico San Matteo, Pavia, Italy; 31 Department of Surgical Sciences, SIUEC Past President, University of Cagliari, Cagliari, Italy; 32 Institute of Clinical Pathology, Department of Laboratory Medicine, Azienda Sanitaria Universitaria Friuli Centrale, Udine, Italy; 33 Endocrinology, Ospedale Unico della Versilia, Azienda USL Toscana Nord Ovest, Pisa, Italy; 34 Endocrinology, Arcispedale S. Maria Nuova IRCCS, ASL Reggio Emilia, Reggio Emilia, Italy; 35 Department of Internal Medicine, Hospital Alto Vicentino (AULSS-7 Veneto), Santorso, VI Italy; 36 Private Practice, Rome, Italy; 37 Facoltà di Medicina e Psicologia, UOS Endocrinologia - UOC Medicina Specialistica Endocrino -Metabolica, AOU Sant’Andrea, Sapienza Università di Roma, Rome, Italy; 38 AME Scientific Committee, Milan, Italy; 39 Functional Department of Endocrinology and Metabolism, AULSS 2 Veneto, Veneto, Italy

**Keywords:** Hypothyroidism, Thyroid, Thyroxine, Triiodothyronine, Desiccated thyroid extracts, Quality of life

## Abstract

**Aim:**

The present guideline (GL) is aimed to improve and standardize the treatment of primary hypothyroidism in non-pregnant adults and to offer all the patients the best possible care across the Italian country.

**Target population:**

Non-pregnant adults with hypothyroidism.

**Excluded population:**

This GL does not cover the treatment of hypothyroidism in children and adolescents under 18 years of age, in women who are pregnant or breastfeeding, nor in subjects with central hypothyroidism. Also patients who require suppressive therapy with levothyroxine after thyroidectomy for thyroid cancer and those with transient iatrogenic hypothyroidism were not considered in this GL.

**Methods:**

The direct costs and the utilization of resources over time were evaluated for the implementation of the appropriate management within the National Health Service. Recommendations were based on the analysis, according to the GRADE methodology, of the evidence from literature. Patients preferences were collected and verified by means of specific bibliographic research and the active participation of two patients’ representatives in the GL development group.

**Results:**

The present GL provides 4 formal graded recommendations and 16 ungraded indications for good clinical practice. An elevated agreement was consistently obtained among the panel members.

**Conclusions:**

The present GL provides operative recommendations—based on the best available evidence and cost-effectiveness analysis—for the treatment of adult patients with primary hypothyroidism. The expected benefits from the dissemination, application and implementation of this GL are the improvement of the quality of care, its homogenization across the national territory and the rationalization of health expenditure in the respect of patient preferences.

**Supplementary Information:**

The online version contains supplementary material available at 10.1007/s40618-025-02652-y.

## Introduction

### Definition, epidemiology and classification

Hypothyroidism is the most common functional alteration of the thyroid gland and is defined as the deficiency of circulating thyroid hormones. Most cases are due to a disease of the thyroid gland and are thus defined as “primary hypothyroidism”, characterized by increased TSH level due to the negative pituitary feed-back induced by the decreased production of thyroid hormones. The maintenance of circulating free thyroxine (FT4) levels within normal range—associated with increased TSH stimulation of the gland—is defined as subclinical hypothyroidism [1].

In a small minority of cases, due to pituitary and/or hypothalamic disorders, hypothyroidism is defined as'central'. In this case, serum TSH is typically low or inappropriately normal; rarely, TSH may be elevated due to secretion of biologically inactive isoforms [2].

Rarely, genetic diseases affecting the thyroid hormone receptors, the biosynthetic or catabolic pathways of thyroid hormones, or the transport proteins of thyroid hormones in the blood or cell membranes are associated with functional changes of thyroid hormones.

Primary hypothyroidism affects approximately 5% of the general population [3], with higher prevalence in females and an increasing incidence with advancing of age.

Currently, in Italy and in other developed countries, severe iodine deficiency is not a relevant cause of hypothyroidism, due to the implementation of iodine supplementation programs [4]. The most frequent causes of acquired primary hypothyroidism are chronic lymphocytic thyroiditis (CLT)—either isolated or, less commonly, associated to other autoimmune diseases—or iatrogenic conditions. These are mostly due to neck surgery, radioiodine treatment or the use of specific drugs [5]. Key interfering drugs include amiodarone, iodine, lithium, interferon, immunologic check-point inhibitors, alemtuzumab, and tyrosine-kinase inhibitors.

### Clinical presentation

Signs and symptoms of hypothyroidism result from the insufficient action of thyroid hormone on target organs. The severity of clinical manifestations directly correlates with the degree and duration of hormone deficiency and may also be influenced by the patient’s age [6].

The most common symptoms and signs are nonspecific and may include: asthenia, fatigability, cold intolerance, constipation, dry skin and pallor, brittle hair, weight gain, periorbital and facial edema, dysphonia, muscle weakness and stiffness, myalgia, menstrual irregularities, reduced mood and memory disorders. Physical examination may reveal pallor, decreased skin elasticity and xerosis, subcutaneous succulence or edema (most commonly in periorbital and pre-tibial areas), bradycardia, and psycho-motor slowing. Hypothyroidism can impact cardiovascular risk, by elevating total cholesterol and LDL fraction. If left untreated, thyroid hormone deficiency may progress to overt hypothyroidism, decline in myocardial contractility and heart rate, dyspnea on exertion, and exercise intolerance. This deterioration, if protracted over time, may result in multi-organ complications, potentially culminating in myxedema coma and death [6].

### Diagnosis

Overt primary hypothyroidism is characterized by serum TSH concentrations exceeding the upper limit of the reference range associated with serum FT4 values below the normal limit. In the most severe forms only, free triiodothyronine (FT3) is reduced as well [6].

The presence of anti-thyroid peroxidase (AbTPO) and anti-thyroglobulin (AbTg) antibodies indicates an autoimmune pathogenesis [7].

To avoid a second blood sampling to determine the free thyroid fractions levels, the “TSH reflex” algorithm is employed. This algorithm automatically measures FT4, and in some Italian districts also TPOAb, on the same blood sample if the TSH level exceeds the pre-established threshold [8]. This approach reduces the costs of the screening without compromising the accuracy of the assessment. The measurement of TSH alone, when it results within the normal range, is sufficient to establish thyroid function as normal. Notably, this initial test modality is inadequate in the rare cases of hypothalamic-pituitary axis pathology or during treatment with drugs that influence TSH secretion. In these patients, it is necessary to measure the free fraction of thyroid hormones: FT4 and, in specific cases only, free triiodothyronine (FT3).

### Treatment and follow-up

Goals of treatment are:

- Restoring euthyroidism through reaching appropriate serum TSH levels;
- Alleviating symptoms and signs of the disease, including the increase of thyroid volume, in specific cases of goitrous hypothyroidism [9, 10];
- Improving patient well-being and quality of life (QoL).

Synthetic LT4, taken orally on an empty stomach, is the most commonly used replacement therapy for hypothyroid patients [9]. Its absorption is good (ranging from 70 to 80% of the ingested dose) and the long plasma half-life of LT4 (about 7 days) results in a constant serum level that may be achieved with a once-daily dosing [11].

LT4 is available in different formulations, including solid or pre-dissolved forms: tablets, soft gel capsules and liquid formulations [12, 13]. To be absorbed tablets require the gastric dissolution phase, so they must be taken on an empty stomach, at least 30–60 min before a meal and at least three hours after food intake. The soft-gel capsules undergo rapid dissolution of the thin gelatin casing with a quick release of the active agent, while liquid formulations do not require gastric disintegration and dissolution. Their absorption is therefore more complete and constant, even in cases of impaired gastric secretion or malabsorption [12–14].

The recommended initial dose in adults with complete loss of thyroid function and without comorbidities is 1.5–1.9 µg/kg/day. This dosage may vary based on patient’s age, lean mass and sex [9, 11]. In the case of mild hypothyroidism, a lower dosage is usually required, which may be subsequently modified based on the clinical response and biochemical normalization.

The prohormone nature of T4 enables the patient's physiological mechanisms to regulate, mainly through peripheral deiodination, the production of T3, the active form of thyroid hormone [15]. However, a minority of hypothyroid patients (in Europe < 10%) report the incomplete resolution of well-being, even when biochemically euthyroid [16]. After the confirmation of normal serum TSH levels it is necessary to carefully rule out the coexistence of other medical conditions, or environmental, psychological or personality problems [16–18]. After their exclusion, a therapeutic trial with the combination of LT4 and LT3 may be considered in selected patients without cardiovascular risk factors [19]. Given the short plasma half-life of T3 (approximately 24 h), the dose should be split into two or three administrations, aiming to maintain the physiological circulating T4/T3 ratio between 13:1 and 16:1.

In Italy the use of dried thyroid extracts is not approved by the Ministry of Health.

The adequacy of the replacement treatment is monitored over time by measuring serum TSH levels possibly associated, in specific circumstances, with FT4 determination [20]. Serum TSH should be maintained within the normal range, generally ranging between 1 and 3 mU/L in adults [21, 22]. The TSH level should be individualized, considering the patient’s age, overall health and well-being. [20]. In elderly individuals (age > 70–75 years), TSH should not be maintained at the lower limit of the normal range, as a moderate but progressive increase in TSH values is commonly observed in the general population with aging [23].

Replacement therapy for hypothyroidism is commonly considered “easy” to manage over time. However, several studies demonstrate that the circulating hormones levels are constantly within the optimal target only in 50% of patients, especially in the elderly [24, 25].

This variability in hormonal stability may be partly explained by clinical conditions and pharmacological interferences that affect LT4 absorption and metabolism. Several clinical conditions and pharmacological agents may interfere with the intestinal absorption or metabolic action of levothyroxine, potentially leading to suboptimal hormonal control despite appropriate dosing. Among the most common clinical scenarios are gastrointestinal disorders (e.g., celiac disease, atrophic gastritis, Helicobacter pylori infection, lactose intolerance, inflammatory bowel disease), which may reduce absorption by altering the intestinal mucosa or gastric pH.

In addition, several medications can interfere with LT4 absorption when taken concomitantly, particularly if spacing is inadequate. These include proton pump inhibitors (PPIs), H2-receptor antagonists, calcium salts, iron supplements, bile acid sequestrants (e.g., cholestyramine), sucralfate, phosphate binders, and certain multivitamin preparations. Moreover, drugs such as antiepileptics (e.g., carbamazepine, phenytoin), rifampicin, and sertraline may enhance hepatic metabolism of thyroid hormones.

A practical summary of the main interfering conditions, mechanisms of action, and management recommendations is provided in Table 1, with the aim of supporting clinical decision-making and appropriate formulation choice.

**Table 1:** Common clinical and pharmacological interferences with LT4 therapy and suggested management

Condition or Drug	Mechanism of Interference	Clinical Impact	Recommended Management
Atrophic gastritis / H. pylori infection	Reduced gastric acidity → impaired tablet dissolution	Reduced LT4 absorption	Consider liquid or soft-gel formulations; treat underlying cause
Celiac disease / IBD	Mucosal inflammation → malabsorption	Fluctuating or inadequate TSH control	Treat underlying condition; consider alternative LT4 forms
Lactose intolerance	Reaction to lactose in excipients	Variable absorption and poor adherence	Use lactose-free LT4 formulations
Proton pump inhibitors (PPIs) / H2 blockers	Increased gastric pH	Decreased tablet bioavailability	Space doses by ≥ 4 h; prefer liquid/soft-gel LT4
Calcium or iron supplements	Chelation of LT4 in GI tract	Impaired absorption	Separate intake by ≥ 4 h
Bile acid sequestrants / sucralfate	Binding LT4 in the gut	Strong reduction in LT4 absorption	Avoid co-administration; adjust timing
Carbamazepine, phenytoin, rifampicin	Induction of hepatic enzymes → increased LT4 clearance	Lower serum FT4 and TSH instability	Monitor TSH closely; increase LT4 dose if needed
Sertraline	Unclear mechanism (possible interaction with thyroid axis)	Variable impact on TSH levels	Monitor thyroid function regularly

Several factors can modify the LT4 requirement. CLT can cause an increased need for LT4 over time due to the progressive decline of thyroid function [26]. Other conditions, sometimes unpredictable, include patient's adherence to therapy, drug interactions, and gastrointestinal diseases [27–29]. In these cases the LT4 dosage can be precisely adjusted based on the TSH values assessed at 4–6-week intervals [9].

Potentially adverse side effects of therapy, particularly in the elderly and in patients with cardiovascular comorbidities, are associated to inappropriately elevated dosage. Iatrogenic thyrotoxicosis can lead to adverse events, including atrial fibrillation in the elderly and osteoporosis in post-menopausal age [30–32], and may also negatively impact on the mood and the quality and duration of life [32].

After identifying the appropriate maintenance dose, TSH dosage should be monitored annually in asymptomatic patients without significant body weight changes or potentially interfering factors [33, 34].

The availability of different LT4 formulations (tablet, soft-gel capsule, and liquid solution) allows for tailored treatment according to individual patient needs.

Tablets require adequate gastric acidity and integrity of the gastrointestinal mucosa to dissolve and be absorbed. Therefore, in the presence of conditions such as atrophic gastritis, Helicobacter pylori infection, or concomitant use of proton pump inhibitors, tablets may be less effective.

Soft-gel capsules have a faster disintegration time and require less gastric acidity, resulting in more predictable absorption in such contexts.

Liquid LT4 formulations are already dissolved and are absorbed independently of gastric pH, showing superior bioavailability in patients with malabsorption syndromes, GI surgery, or complex polytherapy.

For patients with poor adherence or variable routines, formulations with **more flexible timing** (liquid or capsule) may also improve therapeutic stability and patient satisfaction.

### Aim of the guideline

The aim of the present clinical practice GL is to improve the patients care and to support care providers by offering clear recommendations for the most effective and safe treatment of adult patients with primary hypothyroidism.

## Methods

This GL was developed by a task force appointed by the Italian Scientific Societies involved in the field: Associazione Medici Endocrinologi (AME) and Società Italiana di Endocrinologia (SIE) in collaboration with Associazione Italiana di Medicina Nucleare, Imaging Molecolare e Terapia (AIMN), Associazione Italiana della Tiroide (AIT), Associazione Nazionale Infermieri in Endocrinologia e Diabetologia (ANIED), Comitato Associazioni Pazienti Endocrini (CAPE), Federazione delle Associazioni dei Dirigenti Medici Ospedalieri Internisti (FADOI), Società Italiana di Otorinolaringoiatria e Chirurgia Cervico-Facciale (SIOeChCF), and Società Italiana Unitaria di Endocrino-Chirurgia (SIUEC).

The GL was developed according to the Grading of Recommendations Assessment, Development and Evaluation (GRADE) approach [35]. Additionally, we ensured that the contents of the GL were reported in accordance with the AGREE II (Appraisal of Guidelines for REsearch & Evaluation II) checklist [36]. Appendix 1 lists all members of the panel, Evidence Review Team (ERT) and external reviewers who contributed to this GL.

### Clinical question

Recommendations address a central clinical question: What is the most effective and safest treatment for permanent primary hypothyroidism? The focus is on adult patients with autoimmune hypothyroidism or permanent iatrogenic hypothyroidism due to surgery, radiodine, or drugs, such as amiodarone or lithium. The panel formulated the question using the PICO (Population, Intervention, Comparison, Outcome) framework and defined the criteria for study inclusion and exclusion (Appendix 2).

### Selection of outcomes

The panel identified potentially relevant clinical outcomes and rated their importance on a 9-point scale, where 1–3 points indicated outcomes of limited relevance, 4–6 points indicated important but not critical outcomes, and 7–9 points indicated critical outcomes. Only outcomes rated as critical or important were considered in the literature review, with the critical outcomes serving as the basis for formulating recommendations.

### Literature review and assessment of quality of evidence

We conducted a comprehensive systematic literature search on the Cochrane Library, Cochrane Controlled Register of Trials (CENTRAL), MEDLINE, Embase, APA PsycInfo, Web of Science ed Epistemonikos databases from inception to January 2024.

Specific search strategies were used for each database, as specified in each section of Appendix 3. No time or language limits were imposed for all the searches. References of retrieved items were searched for further studies meeting inclusion criteria.

A systematic review was performed through the following steps:

1. Selection of the eligible studies identified from the initial search, based on title and abstract, for retrieval as full text.
2. Identification among retrieved full-text items of relevant studies, based on a priori inclusion and exclusion criteria.
3. Assessment of potential bias using validated instruments such as AMSTAR 2 for systematic reviews [37] and Cochrane tool for randomized controlled trials (RCT) [38].
4. Extraction of main characteristics of the selected studies (patient characteristic, considered outcomes, results), summarized in tables.
5. Outcomes were analyzed by calculating the risk ratio (RR) for dichotomous outcomes and the mean difference or standardized mean difference when the studies all assess the same outcome, but measure it in a variety of ways for continuous variables, both with 95% confidence intervals (CIs). Data synthesis was performed with RevMan 5.4 using fixed effects models.
6. Assessment of heterogeneity by the I^2^ statistic stating the percentage of variability in effects estimated due to heterogeneity rather than chance. The choice between fixed-effect and random-effects models for meta-analyses was based on the degree of clinical and statistical heterogeneity observed. In cases where heterogeneity was minimal (I^2^ < 30%) and the clinical context was homogeneous, fixed-effect models were applied. When heterogeneity was moderate or high (I^2^ ≥ 30%), a random-effects model was used. The I^2^ values and model selection for each outcome are now explicitly reported in the Supplementary Data Table 1.
7. Assessment of the overall quality of available evidence for critical outcomes were rated using the Grading of Recommendations, Assessment, Development, and Evaluation (GRADE) approach [35], categorizing evidence into four levels:
8. High: Reliable data with little likelihood of effect estimates changing with future studies.
9. Moderate: moderately reliable data, whose confidence in estimated effects could be modified by further studies.
10. Low: still limited and uncertain results which need further research for a reliable assessment of the positive and negative effects of the intervention.
11. Very low: available data are not reliable and the estimates of effects should be considered with caution.
12. Synthesis of results were reported in summary of findings and in the Evidence to Decision (EtD) tables, using the GRADEPro Guideline Development tool [39]. EtD tables provides a summary of the results of systematic reviews for desirable and undesirable effects of interventions, quality of available evidence, values and preferences of stakeholders, economic resources needed, equity, acceptability, and feasibility of interventions.
13. GL contents have been reported according to AGREE checklist (The Appraisal of Guidelines for REsearch & Evaluation) [40].

### Pharmacoeconomic studies

The economic evaluation was performed by a pharmacoeconomist with specific expertise (MB).

A survey was performed among the GL panel members, representing various disciplines and regions within the Italian National Health Service (NHS). The survey addressed the specific drivers that contribute to the total cost of hypothyroidism management. Specifically, we investigated type and dosage of the employed drugs, type and quantities of disposable materials, number and time of involvement for each operator, and percentage of patients requiring a caregiver (indirect costs).

We calculated the mean value for each parameter to allow their use in the different regional settings under NHS.

Activity based costing (ABC) analysis was conducted to estimate the expenditures associated to the provision of the different procedures [41]. ABC consists of three steps:

1. Resource identification by means of a specific survey among interdisciplinary panelists. The resources required to implement the procedures under investigation were detailed to quantify each component (time of operators’ activities, materials, drugs dosage, technical resources, etc.).
2. Cost measurement by consultation of scientific literature and specific databases (such as price lists).
3. Results’ valuation: data obtained during the previous steps were combined to define the full cost of each action and of the whole process.

The guideline's economic analysis assessed four major resource categories involved in the procedure under investigation:

- Direct cost paid by NHS for drugs.
- Direct cost paid by NHS for disposable materials.
- Direct cost paid by NHS for operators’ working time and the use of structures.
- Indirect costs sustained by caregivers.

### Development of recommendations

During several web-based meetings, the ERT team presented the following items to the Panel: a summary of the identified studies and their characteristics, a summary of excluded studies along with the reasons for their exclusion, the Evidence to Decision (EtD) framework as a tool for making recommendations, and a report on the economic analysis of treatments related to the clinical question. Subsequently, the panel discussed a draft of the recommendations and voted to judge their strength. Recommendations were categorized as either "strong" or “conditional”, based on considerations such as the balance between effects, certainty of evidence, patients’ values and preferences, economic resources, equity, acceptability, and feasibility of the intervention being considered.

A strong recommendation implies:

- For clinicians: the majority of patients should receive the recommended intervention.
- For patients: almost all properly informed patients should follow the recommendation whereas only a small fraction of them may choose different options.
- For policy makers: the recommendation can serve as a basis for planning and allocating available resources.

A weak recommendation implies:

- For clinicians: the final choice should include a careful consideration of patients’ values and preferences.
- For patients: the majority of properly informed patients will follow the recommendation, but a minority of them may choose different options.
- For policy makers: stakeholders should engage in discussions to address the issue before implementing the recommendation.

If evidence was not available or it was inappropriate for a formal grading of the quality of evidence, the GL panel developed indications for good clinical practice to be used as instructions complementary to recommendations.

### External review

A multi-disciplinary group of experts conducted a comprehensive review of GL draft and provided summary judgments, critiques, and suggestions to improve the document. The Panel evaluated the reviewers' feedback and incorporated the appropriate amendments into the final text, when necessary and relevant.

### Update

The recommendations outlined in this GL will remain valid for a maximum of three years from the publication date. Thereafter, AME will contact the Scientific Societies involved in the production of the document in order to update and revise it.

## Results

### Retrieved literature

The PRISMA flow diagram for the selection of studies is depicted in Appendix 4. Two systematic reviews were identified [42, 43].The methodological quality evaluation of the selected systematic reviews is detailed in Appendix 5.

Fourteen RCTs and no observational study were retrieved. The details of the selected RCTs are showed in Appendix 6.

Pertinent studies were conducted in Croatia [44], Iran [45], Italy [46, 47], Norway [48, 49], Russia (5051), UK [52], USA [53–59]. Most enrolled subjects were women with autoimmune hypothyroidism. Follow-up of the trials ranged between 30 days [53, 54] and 12 months [46, 52].

#### Comparison of LT4 vs. the combination of LT4 and LT3

Ten RCTs were retrieved [44–47, 50–52, 55, 57, 58]. The study design was parallel in all but one, which employed a cross-over [57].

The efficacy in the reduction of fatigue was evaluated in two RCTs [44, 57] (162 participants; follow-up 6–15 weeks) that did not show any significant difference (SMD -0.2, 95% CI from -0.53 to + 0.12; low evidence). The efficacy in the reduction of depression from baseline was evaluated in 4 RCTs [44, 55, 57, 58] (261 participants; follow-up 6 weeks – 4 months) that did not show any significant difference (SMD -0.33, 95% CI from -0.96 to + 0.29; very low evidence). The efficacy in the reduction of somnolence was evaluated in one RCT [44] (135 participants; follow-up 15 weeks) that did not show any significant difference (MD -0.65, 95% CI from -1.7 to + 0.4; low evidence). The efficacy in the reduction of symptoms related to hypothyroidism was evaluated in one RCT [47] by ThyPRO (121 participants; follow-up 6 weeks) that did not show any significant difference (MD + 0.5, 95% CI from -5.29 to + 6.29; very low evidence). The efficacy of change in body weight from baseline was evaluated in 3 RCTs [44–46] (203 participants; follow-up 15 weeks – 12 months) that did not show any significant difference (MD -0.04, 95% CI from -3.42 to + 3.35; moderate evidence). The efficacy of change in TSH levels from baseline was evaluated in 9 RCTs [44–47, 50–52, 55, 58] (321 participants; follow-up 6 weeks – 12 months) that did not show any significant difference (SMD + 0.18, 95% CI from -0.04 to + 0.41; moderate evidence). The efficacy of change in QoL from baseline was evaluated in 1 RCT [58] (41 participants; follow-up 4 weeks) that did not show any significant difference (MD -4, 95% CI from -17.63 to + 9.63; low evidence). The efficacy of change in HDL cholesterol levels from baseline was evaluated in 1 RCT [55] (44 participants; follow-up 4 months) that did not show any significant difference (MD + 1.4, 95% CI from -3.95 to + 6.75; very low evidence).

The efficacy of change in heart rate from baseline was evaluated in 5 RCTs [45, 46, 51, 55, 57] (299 participants; follow-up 15 weeks – 12 months) that showed a significant difference (MD -1.9, 95% CI from -2.48 to -1.31; high evidence). The efficacy of change in FT4 levels from baseline was evaluated in 5 RCTs [44, 46, 47, 52, 55] (243 participants; follow-up 12 weeks – 12 months) that showed a significant difference (MD + 0.27, 95% CI 0.02–0.51; low evidence). The efficacy of change in total cholesterol levels from baseline was evaluated in 5 RCTs [44–47, 51] (239 participants; follow-up 15 weeks – 12 months) that showed a significant difference (MD + 48.22, 95% CI 37.18–59.26; very low evidence). The efficacy of change in LDL cholesterol levels from baseline was evaluated in 3 RCTs [45, 46, 51] (104 participants; follow-up 4–12 months) that showed a significant difference (MD + 12.89, 95% CI 0.89–24.88; moderate evidence).

The complications of treatment, such as subclinical or overt thyrotoxicosis were evaluated in two RCTs [46, 47] at 6 months (145 participants) and in one RCT [46] at 12 months (24 participants) that showed no significant difference (SMD -0.18, 95% CI from -0.1 to + 0.51, and MD + 1.6, 95% CI from -1.75 to + 4.95, respectively) with a low level of evidence.

#### Comparison of LT4 vs. LT3

Two cross-over RCTs in 4 papers were retrieved.

NCT03627611 [48, 49] was conducted in 69 women, with persisting hypothyroid symptoms despite optimal LT4 treatment. Participants were treated with LT4 or LT3 (the dose was one third of the previous LT4 dose) for 12 weeks and then switched to the alternative treatment for other 12 weeks. Fatigue scores were significantly better in the LT3 vs. LT4 treatment. All ThyPRO scores showed an improvement in overall QoL on LT3. Median TSH levels were within the reference range but were lower in the LT4 group compared to the LT3 group and FT4 levels were higher in the LT4 receiving group. LT3 treatment significantly reduced total cholesterol and LDL cholesterol compared to LT4. Among the 47 participants who completed treatment, 34% reported a preference for LT4 therapy, 60% preferred LT3, and 6%. Although the study by Bjerkreim et al. [49] provides interesting data on the perception of well-being in patients treated with LT4 + LT3, its open-label design introduces a high risk of bias, particularly for subjective outcomes. As such, its findings should be interpreted with caution and were rated as low-certainty evidence in the GRADE profile, due to serious limitations in blinding and study design.

In NCT00106119 [53, 54] participants were randomized to receive a regimen of LT3 in three daily pre-meal doses or LT4, with fortnightly dose adjustments, to achieve the target serum TSH level. After a period of at least 30 days to verify achievement of the TSH goal with a constant replacement dose, participants were assigned to the alternative treatment arm with identical follow-up. Overall in the two papers 24 participants completed the study. No difference in TSH values was observed between the two treatment phases. A significant decrease in body weight was observed in the LT3 group. Compared to the LT4 arm, LT3 determined a significant reduction of total and LDL cholesterol, while HDL cholesterol had no significant changes. No difference in QoL was observed between treatments. At the end of the study 8 patients expressed a general preference for LT3 therapy, 9 for LT4 therapy and 7 did not express any preference.

#### Comparison of LT4 vs. desiccated thyroid extract (DTE)

Two cross-over RCTs were retrieved.

In the study by Hoang et al. [56], 79 patients (of whom 70 completed the study) on a stable dose of LT4 for at least 6 months were randomized to receive either LT4 or DTE (the initial dose was chosen using the USP Drug information 2000 conversion table). Drug dosage was adjusted to maintain a TSH level between 0.5 and 3.0 mIU/L. Once the TSH level was in the desired range, patients continued on the assigned treatment for at least 12 weeks before crossing over to the other treatment arm for an additional 16 weeks. None of the patients had a TSH outside the reference range, but TSH levels were higher and FT4 levels lower during DTE treatment. No difference was observed between groups in hypothyroidism symptom scores, general health questionnaires, neuropsychological tests, heart rate, blood pressure, and lipid profile (except for a slightly lower HDL level in the DTE group). There was a significant weight loss on DTE therapy compared to LT4. At the end of the study, 49% preferred treatment with DTE, 19% with LT4 and 33% did not express any preference.

In the study by Shakir et al. [59] 99 patients on a stable dose of LT4 for at least 6 months were randomized to LT4, LT4 + LT3, or DTE (22 weeks for each treatment) and 75 completed the study. Here we report only results for the comparison between LT4 and DTE. The dose was adjusted to maintain TSH levels between 0.27 and 4.20 mIU/L. Mean serum TSH remained within reference range with minimal variation, although levels were slightly higher in patients treated with DTE. Mean FT4 levels at the end of the study were 1.44 ng/dL in the LT4 group vs. 0.98 ng/dL in the DTE group. No difference was observed in QoL, body weight, total cholesterol, LDL cholesterol and HDL cholesterol. Heart rate was minimally increased in the DTE arm. At the end of the study no significant differences were observed between the groups as for treatment preference.

### Economic evaluation

The preliminary survey conducted among panel members pointed out that the group of patients experiencing difficulty in normalizing biochemical parameters or expressing dissatisfaction with their treatment accounts for approximately 18% of cases (Table 2).

**Table 2:** Stratification of hypothyroid patients according to disease course and treatment

Distribution	Mean
Patients with standard course	79.82%
Patients with non-standard course	18.89%
Patients treated with LT3 alone	0.018%
Patients treated with LT4 + LT3 combination	1.27%
Patients treated with DTE	0%
Total	100%

Table 3 provides an overview of the costs associated with the initial management framework for patients with hypothyroidism.

**Table 3:** Average cost of the initial framework

	Mean	Minimum	Maximum
Procedures for the diagnosis of hypothyroidism	€ 120.89	€ 66.50	€ 193.75
Cost of health operators	€ 14.88	€ 11.42	€ 18.33
Total cost of the initial framework	€ 135.77	€ 77.92	€ 212.08

However, a significant proportion of patients, approximately one-third, suffer from hypothyroidism as a result of surgery [60]. For these patients the initial assessment cost is minimal and the therapy is started immediately after the operation. Thus, the weighted average cost for the initial assessment is slightly less than € 100.

Table 4 reports the annual cost of the patient with hypothyroidism and standard course.

**Table 4:** Overall annual cost of the hypothyroid patient with standard course

	General		Males		Females	
	Mean	Min–Max	Mean	Min–Max	Mean	Min–Max
Drugs	**€ 48.44**	€ 40.39–56.49	**€ 56.35**	€ 46.40–66.31	€ **43.13**	€ 35.76–50.50
Follow-up	**€ 61.71**	€ 24.79–100.80	**€ 61.71**	€ 24.79–100.80	**€ 61.71**	€ 24.79–100.80
Personnel	**€ 11.44**	€ 9.86–13.02	**€ 11.44**	€ 9.86–13.02	**€ 11.44**	€ 9.86–13.02
Total of direct costs	**€ 121.59**	€ 75.04–170.31	**€ 129.51**	€ 81.05–180.13	**€ 116.28**	€ 70.41–164.32
Indirect costs	**€ 17.16**	€ 8.62–25.70	**€ 17.16**	€ 8.62–25.70	**€ 17.16**	€ 8.62–25.70
**Total cost**	**€ 138.75**	€ 83.65–196.01	**€ 146.66**	€ 89.66–205.83	**€ 133.44**	€ 79.02–190.02

Table 5 reports the annual cost of the patient with hypothyroidism and non-standard course.

**Table 5:** Overall annual cost of the hypothyroid patient with non-standard course

	General		Males		Females	
	Mean	Min–Max	Mean	Min–Max	Mean	Min–Max
Drugs	**€ 92.23**	€ 79.39–105.07	**€ 100.75**	€ 87.55–113.95	€ **87.64**	€ 73.51–101.78
Follow-up	**€ 112.18**	€ 50.48–166.97	**€ 112.18**	€ 50.48–166.97	**€ 112.18**	€ 50.48–166.97
Personnel	**€ 20.12**	€ 14.53–25.78	**€ 20.12**	€ 14.53–25.78	**€ 20.12**	€ 14.53–25.78
Total of direct costs	**€ 224.53**	€ 144.40–297.82	**€ 233.05**	€ 152.56–306.70	**€ 219.95**	€ 138.53–294.53
Indirect costs	**€ 25.85**	€ 14.40–37.30	**€ 25.85**	€ 14.40–37.30	**€ 25.85**	€ 14.40–37.30
Total cost	**€ 250.38**	€ 158.80–335.12	**€ 258.90**	€ 166.97–344.00	**€ 245.80**	€ 152.93–331.83

Table 6 reports the annual cost of the patient with hypothyroidism treated with drugs other than LT4.

**Table 6:** Overall annual cost of the hypothyroid patient on treatment with drugs other than LT4

	General		Males		Females	
	Mean	Min–Max	Mean	Mean	Min–Max	Mean
Drugs	**€ 206.54**	€ 206.54–206.54	**€ 218.03**	€ 218.03–218.03	€ **192.21**	€ 192.21–192.21
Follow-up	**€ 87.36**	€ 51.52–123.33	**€ 87.36**	€ 51.52–123.33	**€ 87.36**	€ 51.52–123.33
Personnel	**€ 20.14**	€ 16.19–24.09	**€ 20.14**	€ 16.19–24.09	**€ 20.14**	€ 16.19–24.09
Total of direct costs	**€ 314.04**	€ 274.20–352.70	**€ 325.53**	€ 285.69–364.20	**€ 299.71**	€ 259.87–338.38
Indirect costs	**€ 14.77**	€ 7.52–22.02	**€ 14.77**	€ 7.52–22.02	**€ 14.77**	€ 7.52–22.02
Total cost	**€ 328.81**	€ 281.77–375.98	**€ 340.30**	€ 293.21–386.22	**€ 314.48**	€ 267.39–360.40

Tables 7 and 8 report the overall annual cost of patients with hypothyroidism in Italy.

**Table 7:** Overall annual cost for the management of the patient with primary hypothyroidism

	General		Males		Females	
	Mean	Min–Max	Mean	Min–Max	Mean	Min–Max
Initial framework	**€ 135.77**	€ 77.92–212.08	**€ 135.77**	€ 77.92–212.08	**€ 135.77**	€ 77.92–212.08
Weighted initial framework (A)	**€ 89.61**	€ 51.42–139.97	**€ 89.61**	€ 51.42–139.97	**€ 89.61**	€ 51.42–139.97
Patients with standard course (79.82%)						
Overall cost	**€ 138.75**	€ 83.65–196.01	**€ 146.66**	€ 89.66–205.83	**€ 133.44**	€ 79.02–190.02
Weighted overall cost (B)	**€ 110.75**	€ 66.78–156.46	**€ 117.07**	€ 71.57–164.30	**€ 106.52**	€ 63.08–151.68
Patients with non-standard course (18.89%)						
Overall cost	**€ 250.38**	€ 158.80–335.12	**€ 258.90**	€ 166.97–344.00	**€ 245.80**	€ 152.98–331.83
Weighted overall cost (C)	**€ 47.29**	€ 29.99–63.29	**€ 48.90**	€ 31.53–64.97	**€ 46.42**	€ 28.88–62.67
Patients on treatment with drugs other than LT4 (1.29%)						
Overall cost	**€ 328.81**	€ 281.77–375.98	**€ 340.30**	€ 293.26–387.48	**€ 314.48**	€ 267.45–361.66
Weighted overall cost (D)	**€ 4.24**	€ 3.64–4.85	**€ 4.39**	€ 3.78–5.00	**€ 4.06**	€ 3.45–4.67
Annual average cost (A + B + C + D)	**€ 251.89**	€ 151.83–364.58	**€ 259.97**	€ 158.31–374.00	**€ 246.60**	€ 146.84–358.99

**Table 8:** Overall annual cost for the management of the patient with primary hypothyroidism excluding personnel costs

	Mean	Minimum	Maximum
Initial framework	€ 120.89	€ 66.50	€ 193.75
Weighted initial framework (A)	**€ 79.79**	€ 43.89	€ 127.88
Patients with standard course (79.82%)			
Overall cost	**€ 127.31**	€ 73.79	€ 182.99
Weighted overall cost (B)	**€ 101.62**	€ 58.91	€ 146.07
Patients with non-standard course (18.89%)			
Overall cost	**€ 230.26**	€ 144.27	€ 309.33
Weighted overall cost (C)	**€ 43.49**	€ 27.25	€ 58.42
Patients on treatment with drugs other than LT4 (1.29%)			
Overall cost	**€ 308.67**	€ 265.57	€ 351.89
Weighted overall cost (D)	**€ 3.98**	€ 3.43	€ 4.54
Annual average cost (A + B + C + D)	**€ 228.88**	€ 133.47	€ 336.91

## Recommendations and indications for good clinical practice

Replacement therapy is essential for patients with hypothyroidism. The treatment should achieve and sustain over time: 1) the resolution of symptoms and signs of the disease, 2) the normalization of serum TSH and FT4 levels, 3) the improvement in QoL.

Table 9 shows the recommendations agreed on by the panel about the clinical question: “What is the most effective and safest treatment for permanent primary hypothyroidism in adults?”.

**Table 9:** List of recommendations for adult patients with primary hypothyroidism

#	Quality of evidence	Recommendations	Strength of recommendation
1	Very Low	The panel recommends monotherapy with LT4 in comparison to combined therapy with LT4 + LT3	Strong, in favor of the intervention
2	Very Low	The panel suggests the administration of oral LT4 on an empty stomach	Conditional, in favor of the intervention
3	Very Low	In patients whose hypothyroidism is due to previous total thyroidectomy, the panel suggests an initial dose of LT4 adequate to completely replace the loss of thyroid function. This generally ranges between 1.5 and 1.9 µg/kg of body weight/day. Age and gender influence the dosage that should be adjusted according to the patient's conditions	Conditional, in favor of the intervention
4	Very Low	The panel suggests the measurement of serum TSH 4–6 weeks after the start of replacement therapy, to assess its adequacy	Conditional, in favor of the intervention

Indications for good clinical practice, complementary to the previous formal recommendations, have been formulated in accordance with the methodological manual of the national GL system. They provide operational messages on specific aspects for which no direct comparison studies between the different therapeutic options are available. These indications, based on the extensive clinical experience of the GL panelists, received their full agreement. For these reasons, they are presented as a guidance for good healthcare practice.

1. The use of the different formulations of LT4 currently available (tablets, liquid or soft-gel capsules) should be considered based on patient's preference, lifestyle habits and presence of comorbidities. In general, they are equally effective for the purposes of replacement therapy [61, 62] (ungraded).
2. LT4 therapy should be preferably taken in the morning, at least 30 min — ideally 60 min — before the intake of food or beverages to optimize hormone absorption [63–65] (ungraded).
3. LT4 therapy should be taken at an adequate time interval from the drugs potentially interfering with its absorption [66–70] (ungraded). Among these it is worthwhile mentioning proton pump inhibitors and other gastro-protectors, calcium and iron salts, and bile acid sequestrants.
4. In patients who are unable to take LT4 in the morning, administration at different times, particularly late in the evening, can be considered. A fasting period of at least three hours should be observed beforehand as the absorption of LT4 depends on the gastric content acidity and is impaired by the food content of the stomach [71–73] (ungraded).
5. Serum TSH should be monitored 4–6 weeks after any change of the dosage or formulation of LT4. Once the adequate replacement dosage has been found, monitoring is recommended after 6 months and then at 12-month intervals or more frequently as clinically indicated. Dosage adjustments may be necessary in case of weight changes, in the elderly, if internal medicine comorbidities occur or if drugs interfering with absorption are introduced [62, 74, 75] (ungraded).
6. If the replacement treatment is effective, both biochemically and clinically, it is advisable to maintain the patient with a constant dosage and formulation over time [74] (ungraded).
7. In the replacement therapy of hypothyroidism it is not advisable the use of desiccated thyroid or the therapy with LT3 alone [48, 49, 54, 56, 59] (ungraded).
8. In the elderly, frail patients, and subjects with cardiovascular disease or significant comorbidities, regardless of the etiology and severity of hypothyroidism, it is advisable to start the replacement therapy cautiously with low doses. Afterwards, the dose should be gradually modified based on serum TSH values, symptom control and absence of side effects. In elderly patients, it is advisable to maintain TSH levels at the upper limits of normal range [76, 77] (ungraded).
9. Follow-up of hypothyroidism is based on serial evaluation of TSH and FT4, while it is not useful to routinely measure FT3 [78] (ungraded).
10. In subjects with overt hypothyroidism induced by the therapy with amiodarone, the anti-arrhytmic drug can be continued in presence of a defined cardiological indication. Treatment with LT4 should be started cautiously with gradual dose, to avoid a therapeutic over-dosage and the worsening of the risk of arrhythmia [79, 80] (ungraded).
11. In subjects starting lithium therapy, due to the high frequency of incident hypothyroidism (especially in females), thyroid function should be monitored at 6–12-month intervals. If hypothyroidism occurs, LT4 replacement treatment should be started without withdrawing lithium therapy [81] (ungraded).
12. Pre-menopausal women on replacement therapy for hypothyroidism should be informed about the need for medical consultation in case of diagnosis or planning of pregnancy, for early dosage adjustment [82–84] (ungraded).
13. In elderly subjects with TSH not yet at target but with values below 10 mIU/L, the patient's overall conditions should be carefully evaluated before increasing the dose of LT4. The available evidence demonstrate that the normalization of TSH to the target levels of general population benefits lipid profile but has limited impact on the major clinical outcomes [85, 86] (ungraded).
14. There is no strong evidence that treatment of subclinical hypothyroidism (elevated TSH associated with normal FT4 values) provides significant clinical benefits [87, 88]. However, in case of persistent subclinical hypothyroidism on LT4 replacement therapy, normalization of serum TSH is advisable (ungraded).
15. After the diagnostic phase and the achievement of stable target hormonal values, routine management of the patient with hypothyroidism can be safely performed by the general practitioner (GP) (ungraded).
16. Patients with hypothyroidism who are managed by the GP take advantage of endocrinological consultation in the following specific clinical conditions (ungraded):
17. Planning of pregnancy, diagnosis of pregnancy, postpartum and breastfeeding period;
18. Difficulty in maintaining TSH values within the reference limits;
19. Persistence of symptoms attributed to hypothyroidism despite the achievement of biochemical euthyroidism (serum TSH consistently within the reference range);
20. Occurrence of further pathologies that may complicate the clinical picture;
21. Therapy with drugs that modulate the immune system and/or influence thyroid function (e.g., interferon, alemtuzumab, amiodarone).

## Discussion

Thyroid replacement treatment is usually considered as an easy task but is sometimes unsatisfactory in the clinical practice. Patients' dissatisfaction due to the persistence of ill-defined symptoms or physicians' hindrance because of a recurrently umbalanced thyroid hormone profile testify that treament of hypothyroidism may be challenging. This issue remains unchanged despite the several hypothyroidism GL released for clinical practice and in spite of the good level of adherence to their recommendations reported by Italian endocrinologists [33, 89].

### Why upgrading recommendation in presence of weak evidence

The panel of authors, despite the weak evidence in favor of treatment with LT4 alone in comparison to combination treatment with LT4 and LT3, unanimously provided a “strong recommendation”. Actually, the combination treatment with LT4 and LT3 results only in minor advantages in the level of total and LDL cholesterol while does not induce significantly positive effects on:

- Control of symptoms and signs related to hypothyroidism;
- Maintenance of body weight;
- Normalization of peripheral thyroid hormone profile;
- Patients’ QoL.

### Pathophysiological considerations supporting the upgrade:

- The diagnosis of hypothyroidism is based on the determination of circulating TSH and FT4 levels only. LT4 is the main hormone produced by the thyroid while only a small portion of circulating T3 is produced directly by the thyroid. Its majority is synthesized peripherally, in the pituitary, liver, kidneys, and target cells [90].
- LT4 is a prohormone that is converted into the active hormone (T3) by peripheral deiodinases. The expression of activating deiodinases and their activity is influenced by circulating L-thyroxine concentrations. Thus, even slight elevations of circulating T4 decrease the conversion of T4 into T3 and prevent the exposure of tissues—most importantly the cardiovascular system—to an inappropriate activity of thyroid hormones [90]. Also, in conditions of iodine excess the increased availability of circulating LT4 is not paralleled by T3 elevation. For instance, the heavily iodinated amiodarone inhibits the activation of deiodinase and initially causes the reduction of T3 levels. These changes are followed by the achievement of a steady state with T3 levels close to the lower threshold of normal range [91].
- The measurement of circulating FT3 is less reliable than that of FT4 and TSH. This drawback is due to the peripheral source of production of T3. Most T3 is metabolized locally and the measurement of circulating T3 is not completely reliable, making difficult the appropriate monitoring of combined LT4/LT3 therapy [92, 93].
- In patients with chronic comorbidities or intercurrent acute diseases, the measurement of circulating T3 may not reflect the functional state of the thyroid due to the “low T3 syndrome” occurring in these conditions [94, 95].

### Clinical management considerations supporting the upgrade:

e. The short half-life of the T3 molecule makes less manageable the combined therapy in clinical practice. The optimal treatment with T3 would require three daily administrations, while a single daily administration of LT4 is adequate. Multiple daily intakes negatively influence the adherence to therapy, a relevant problem for the reliable management of chronic disease with long life expectancy [96, 97].
f. Studies on treatment adherence performed in subjects with hypothyroidism confirm that this treatment is consistently adequate in less than 50% of patients, especially in patients who are elderly or on multi-drug treatment. In over 2/3 of patients who are not at target, a condition of subclinical iatrogenic thyrotoxicosis may insidiously occur with potential detrimental consequences [24, 98, 99].

### Good clinical practice and international GL support to the upgrade:

g. The major Scientific Societies—in Italy, Europe and the USA—strongly recommend the treatment of hypothyroidism with LT4 alone in non-pregnant adults. An upgrade of the moderate-weak level of evidence is generally made [9, 33, 100].
h. In the 2012 European Thyroid Association GL, which evaluated the clinical conditions in which the combined LT4 + LT3 therapy may be useful, therapy with LT4 alone is recommended as standard initial therapy [19].
i. A recent extensive survey, involving over 6000 European specialists with specific expertise in the field of thyroid disease in 28 countries, confirmed that in clinical practice the initial therapy of choice for hypothyroidism is LT4 alone [101].

### Economic considerations

The overall annual cost of the hypothyroid patients in Italy was calculated on the basis of data acquired by a survey among the panel members that allowed the stratification of the patients according to their treatment and course, and by the definition of the associated expenses.

In Italy, over the last 20 years, an average of approximately 40,000 thyroidectomies (both total and partial) have been performed each year, approximately 10,000 of which were due to thyroid malignancy [60]. Since benign nodular disease, that is the primary indication for surgery, is often bilateral, total thyroidectomy is commonly indicated [102]. Lobo-isthmectomy either for benign or malignant disease is still performed in a minority of patients in Italy [103]. In addition, after lobo-isthmectomy, a sizeable number of patients require LT4 treatment [104]. The percentage of patients who have undergone surgery and are not treated with LT4 can therefore be considered negligible.

In this analysis, we defined a *“standard course”* as the therapeutic pathway of a clinically stable patient treated with LT4 tablets, showing good adherence, absence of interfering medications or comorbidities, and requiring only routine follow-up.

A *“non-standard course”* was defined as any scenario requiring additional monitoring, frequent TSH testing, dose adjustments, or the use of alternative formulations (e.g., soft-gel capsules or liquid LT4) due to poor absorption, drug interactions, or other clinical complexit.

Assuming a standard course for all patients, the overall cost for the NHS of the annual follow-up for the management of hypothyroidism—based on drugs, operators, investigations and indirect costs—can be calculated in approximately € 417 million. This calculation is based on a 5% prevalence of hypothyroidism in the general population (i.e. 3 million individuals out of a total Italian population of 60 million) multiplied by an estimated annual individual cost of € 139. However, the actual management of hypothyroidism is more complex and numerous factors may influence its costs. The costs associated to biochemical, clinical and/or instrumental monitoring are highest for fragile patients and for those with comorbidities. These include cardiovascular patients with arrhythmias or coronary artery disease, individuals undergoing therapy with interfering drugs such as amiodarone, lithium, or oncological medications, and subjects with gastrointestinal disturbances (pediatric and adolescent patients and women planning or undergoing pregnancy are out the scope of this GL). The cost of follow-up is also influenced by the duration of the disease: in all chronic diseases, the frequency of check-ups and any therapeutic change is greater during the first years while subsequently a more inertial approach gradually supervenes [105]. This clinical approach initially leads to a reduction in costs but may result in adverse clinical outcomes over the long term [106]. Therefore, systematic clinical check-ups should be carried out even if the therapeutic changes appear modest.

Based on a survey among the panel members, it has been estimated that the group of hypothyroid patients with non-standard course represents approximately 18% of the total population of patients with hypothyroidism. This estimate may be incorrect: the panel members are opinion leaders of the participating Scientific Societies and may not represent the average operator in the specialty to which they belong. Furthermore, patients who are more difficult to manage may be preferentially sent to the reference centers where they operate.

The increase in costs for hypothyroid patients with a non-standard course is due to the doubling of the costs of all items considered (drugs used and frequency of controls, with a consequent increase in the costs of investigations, health workers and indirect costs). The increase in costs is primarily due to the rise in monitoring requirements and, to a lesser extent, the cost of medications. However, in over 60% of cases within this category, newer formulations are preferred due to their potential to enhance patient adherence and provide more consistent absorption. Nevertheless, these benefits come with higher associated costs. Before prescribing these formulations, a careful anamnestic investigation is appropriate: when concomitant pathologies or therapies predict an altered absorption of the tablets or, given lifestyle habits, failure to comply with fasting, the use of liquid or soft-gel formulations should be considered. In these patients, the cost of the drug would increase, but the increase in direct and indirect costs—related to more frequent laboratory and clinical checks—and the occurrence of patient stress may be prevented.

Although alternative LT4 formulations (such as soft-gel capsules and liquid solutions) are more expensive than standard tablets, their use in selected clinical conditions may result in an overall reduction of healthcare costs. In patients with absorption impairments or pharmacological interferences, switching to a more bioavailable formulation may lead to more stable TSH values and consequently reduce the need for frequent follow-up visits and laboratory testing. Furthermore, improved adherence and patient satisfaction may help prevent repeated dosage adjustments and unnecessary specialist consultations.

Therefore, in the presence of clear clinical or pharmacological predictors of altered LT4 absorption, the higher drug cost may be offset by reduced indirect and follow-up-related costs, supporting a cost-effective, personalized approach to hypothyroidism management.

In the few patients treated with therapies alternative to LT4, the significant increase in annual cost is almost entirely due to the high cost of the drugs. The higher cost, in association with the greater complexity of the therapy (LT3 must be divided into 2–3 daily doses necessitating more frequent and intensive monitoring) and the clinical risk due to the administration of an already active hormone which escapes normal homeostatic mechanisms, does not make this treatment clinically convenient. Thus, a trial of combined treatment should be preferentially considered in the rare conditions characterized by reduced activity of peripheral deiodinases or transport mechanisms [53].

In this GL, the recommendations emerging from the evaluation of specific papers and the suggestions of good clinical practice of the panel members may produce a more careful cost management. Although hypothyroidism is an overall inexpensive pathology (the overall individual cost for 80% of patients is on average € 139/year), the relevant prevalence of the pathology – up to 5% of the general population—and the many years of treatment—on average 40—lead to a high overall cost that needs a careful management.

### Conclusions

Good clinical practice indications, based on the methodological analysis of the reviewed studies, are complementary to the recommendations for hypothyroidism in order to achieve two primary goals: the optimization of the use of drugs and their formulations for the management of a chronic disease, and the effective and widespread application in clinical settings. The choice of the appropriate formulation of LT4 should consider the characteristics of the patient, like his/her compliance, concomitant intake of other drugs, comorbidities—especially in case of interference with intestinal absorption—and patient’s preference. At the same time costs should be contained choosing the treatment with the best cost-effectiveness ratio [24, 98, 107]. In specific cases with a non-standard course, the use of more expensive LT4 formulations could be advantageous due to the reduction of the laboratory and clinical controls, the decrease of indirect costs, and the prevention of patients dissatisfaction. The indication remains, regardless of costs, for the use of LT3 in addition to LT4 for the small minority of patients who, after the attainment of biochemical euthyroidism with LT4 alone, do not achieve subjective clinical compensation in terms of perception of psycho-physical well-being. Moreover, recent insights suggest that interindividual differences in tissue responsiveness to thyroid hormone may partly explain the persistence of symptoms in biochemically euthyroid patients. In particular, genetic polymorphisms affecting thyroid hormone transporters, deiodinases (especially DIO2), or nuclear receptors have been proposed as potential contributors to this phenomenon. These variants may impair intracellular T3 availability or signaling, leading to reduced tissue-level euthyroidism despite normal serum TSH levels. Although such polymorphisms are not routinely assessed in clinical practice, their presence may provide a pathophysiological basis for residual symptoms in a subset of patients. Nevertheless, in the absence of documented genetic variants, their existence alone does not justify a systematic use of combination therapy with LT4 and LT3. This emerging area of research, however, warrants consideration when evaluating difficult-to-treat cases [108]. Due to the potential deficiency of peripheral deiodinase or intracellular transportation – that are difficult to be assessed in the clinical practice—in such cases a trial of combined therapy with LT3 + LT4 may be appropriate. The clinical benefit and the potential side effects should be monitored and verified over time.

The recommendations included in this GL are not likely to lead to an increase in the costs of management of hypothyroidism. For non-pregnant hypothyroid adults, the recommendation is to start therapy with LT4 alone—recommendation unanimously upgraded to “strong”—the therapeutic approach that is most economical and requires less intense monitoring.

The economic evaluation confirms that the panel approach in subjects with a non-standard course allows, despite the prescription of more expensive LT4 formulations, adequate efficacy in specific clinical contexts, due to greater patient adherence (single-dose intake) and interruption of the more intensive monitoring. The use of combined therapy with LT4 + LT3 in the above specified cases, would lead to an increase of at least 20% in the annual expenditure due to the cost of drugs, compared to the use of pre-dissolved forms of LT4, including the most expensive soft-gel capsules.

Unfortunately, an element of social inequality could persist in specific cases, due to the use of soft-gel formulations of LT4 that are at full expense of the patients, at variance with all other formulations that are reimbursed by the NHS.

The widespread adoption of the GL recommendations and good clinical practice indications—tailored to the realities of the Italian healthcare system—could promote an optimal and more uniform approach for the management of hypothyroidism across the national territory.

## Supplementary Information

Below is the link to the electronic supplementary material.

Supplementary file1 (DOCX 138 KB)

## Abbreviations

ABC: Activity based costing
AbTg: Anti-thyroglobulin antibodies
AbTPO: Anti-thyroid peroxidase antibodies
AGREE: The Appraisal of Guidelines for research & Evaluation
AIFA: Agenzia Italiana del Farmaco (Italian agency for drugs)
AIMN: Associazione Italiana di Medicina Nucleare, Imaging Molecolare e Terapia
AIT: Associazione Italiana della Tiroide
AME: Associazione Medici Endocrinologi (Italian Association of Clinical Endocrinologists)
AMSTAR: Assessing the Methodological Quality of Systematic Reviews
ANIED: Associazione Nazionale Infermieri in Endocrinologia e Diabetologia
AMSTAR: A MeaSurement Tool to Assess systematic Reviews
BDI: Beck Depression Inventory
BMI: Body mass index
CAPE: Comitato Associazioni Pazienti Endocrini
CES-D: Comprehensive Epidemiological Screens for Depression
CI: Confidence interval
CLT: Chronic lymphocytic thyroiditis
CTX: C-terminal telopeptide of type 1 collagen
CV: Cardiovascular
CVLT: California Verbal Learning Test
DOI: Digital Object Identifier
DTE: Desiccated thyroid extract
ECG: Electrocardiogram
ENT: Ear, nose, and throat
ERT: Evidence review team
EtD: Evidence to Decision
FADOI: Federazione delle Associazioni dei Dirigenti Medici Ospedalieri Internisti
FT3: Free triiodothyronine
FT4: Free thyroxine
GHQ-28: Goldberg’s general Health Questionnaire
GHQ-30: General Health Questionnaire-30
GL: Guideline
GP: General practitioner
GRADE: Grading of Recommendations Assessment, Development and Evaluation
HADS: Hospital Anxiety and Depression questionnaire
HDL: High-density lipoprotein
LDL: Low-density lipoprotein
LT3: Levotriiodothyronine
LT4: Levothyroxine
Max: Maximum
MCT: Memory Comparison Task
MESH: Medical Subject Headings
MFI-20: Multidimensional Fatigue Inventory
Min: Minimum
MOS: Medical Outcomes Study
NHS: National Health System
PICO: Population, Intervention, Comparison, Outcome
POMS: Profile of Mood States
PRISMA: Preferred Reporting Items for Systematic reviews and Meta-Analyses
QoL: Quality of life
RAND-36: Mental health and vitality subscales of the Rand 36-item health survey
RBMT: Rivermead Behavioral Memory Test
RCT: Randomized controlled trial
RR: Relative risk
rT3: Reverse T3
SCL-90: Symptom Checklist-90
SHBG: Sex hormone-binding globulin
SIE: Società Italiana di Endocrinologia
SF-36: Short form 36
SIOeChCF: Società Italiana di Otorinolaringoiatria e Chirurgia Cervico-Facciale
SIUEC: Società Italiana Unitaria di Endocrino-Chirurgia
SMD: Standardized mean difference
T3: Triiodothyronine
T4: Thyroxine
Tg: Thyroglobulin
TSH: Thyroid-stimulating hormone
TSQ: Thyroid symptom questionnaire
ThyPRO: Thyroid-specific patient reported outcome
VAS: Visual analogue scale
WAIS: Wechsler Adult Intelligence Scale
WHO: World Health Organization
WMS-III: Wechsler memory scale

## References

[1] Devdhar M, Ousman YH, Burman KD (2007) Hypothyroidism. Endocrinol Metab Clin North Am 36:595–615. 10.1016/j.ecl.2007.04.008 17673121 10.1016/j.ecl.2007.04.008

[2] Beck-Peccoz P, Rodari G, Giavoli C, Lania A (2017) Central hypothyroidism - a neglected thyroid disorder. Nat Rev Endocrinol 13:588–598. 10.1038/nrendo.2017.47 28549061 10.1038/nrendo.2017.47

[3] Garmendia Madariaga A, Santos Palacios S, Guillén-Grima F, Galofré JC (2014) The incidence and prevalence of thyroid dysfunction in Europe: a meta-analysis. J Clin Endocrinol Metab 99:923–931. 10.1210/jc.2013-2409 24423323 10.1210/jc.2013-2409

[4] De Angelis S, Medda E, Rotondi D, Masocco M, Minardi V, Contoli B et al (2024) Fifteen years of iodine prophylaxis in Italy: results of a nationwide surveillance (period 2015–2019). J Clin Endocrinol Metab 109:e495–e507. 10.1210/clinem/dgad593 37820735 10.1210/clinem/dgad593 PMC10795908

[5] Burch HB (2019) Drug effects on the thyroid. N Engl J Med 381:749–761. 10.1056/NEJMra1901214 31433922 10.1056/NEJMra1901214

[6] Chaker L, Bianco AC, Jonklaas J, Peeters RP (2017) Hypothyroidism. Lancet 390:1550–1562. 10.1016/S0140-6736(17)30703-1 28336049 10.1016/S0140-6736(17)30703-1 PMC6619426

[7] Dwivedi SN, Kalaria T, Buch H (2023) Thyroid autoantibodies. J Clin Pathol 76:19–28. 10.1136/jcp-2022-208290 36270794 10.1136/jcp-2022-208290

[8] Taher J, Brinc D, Gilmour JA, Beriault DR (2019) Validating thyroid-stimulating hormone (TSH) reflexive testing cutpoints in a tertiary care institution. Clin Chem Lab Med 58:e11–e13. 10.1515/cclm-2019-0396 31339854 10.1515/cclm-2019-0396

[9] Jonklaas J, Bianco AC, Bauer AJ, Burman KD, Cappola AR, Celi FS et al (2014) Guidelines for the treatment of hypothyroidism: prepared by the American Thyroid Association task force on thyroid hormone replacement. Thyroid 24:1670–1751. 10.1089/thy.2014.0028 25266247 10.1089/thy.2014.0028 PMC4267409

[10] Hegedüs L, Hansen JM, Feldt-Rasmussen U, Hansen BM, Høier-Madsen M (1991) Influence of thyroxine treatment on thyroid size and anti-thyroid peroxidase antibodies in Hashimoto’s thyroiditis. Clin Endocrinol (Oxf) 35:235–238. 10.1111/j.1365-2265.1991.tb03528.x 1742880 10.1111/j.1365-2265.1991.tb03528.x

[11] Fish LH, Schwartz HL, Cavanaugh J, Steffes MW, Bantle JP, Oppenhjeimer JH (1987) Replacement dose, metabolism, and bioavailability of levothyroxine in the treatment of hypothyroidism. Role of triiodothyronine in pituitary feedback in humans. N Engl J Med 316:764–770. 10.1056/NEJM198703263161302 3821822 10.1056/NEJM198703263161302

[12] Nagy EV, Perros P, Papini E, Katko M, Hegedüs L (2021) New formulations of levothyroxine in the treatment of hypothyroidism: trick or treat? Thyroid 31:193–201. 10.1089/thy.2020.0515 33003978 10.1089/thy.2020.0515

[13] Liu H, Li W, Zhang W, Sun S, Chen C (2023) Levothyroxine: conventional and novel drug delivery formulations. Endocr Rev 44:393–416. 10.1210/endrev/bnac030 36412275 10.1210/endrev/bnac030 PMC10166268

[14] Sachmechi I, Lucas KJ, Stonesifer LD, Ansley JF, Sack P, Celi FS et al (2023) Efficacy of levothyroxine sodium soft gelatin capsules in thyroidectomized patients taking proton pump inhibitors: an open-label study. Thyroid 33:1414–1422. 10.1089/thy.2023.0382 37885233 10.1089/thy.2023.0382 PMC10754356

[15] Jonklaas J, Davidson B, Bhagat S, Soldin SJ (2008) Triiodothyronine levels in athyreotic individuals during levothyroxine therapy. JAMA 299:769–777. 10.1001/jama.299.7.769 18285588 10.1001/jama.299.7.769

[16] Perros P, Van Der Feltz-Cornelis C, Papini E, Nagy EV, Weetman AP, Hegedüs L (2023) The enigma of persistent symptoms in hypothyroid patients treated with levothyroxine: a narrative review. Clin Endocrinol (Oxf) 98:461–468. 10.1111/cen.14473 33783849 10.1111/cen.14473

[17] Perros P, Hegedüs L, Nagy EV, Papini E, Hay HA, Abad-Madroñero J et al (2022) The impact of hypothyroidism on satisfaction with care and treatment and everyday living: results from e-mode patient self-assessment of thyroid therapy, a cross-sectional, international online patient survey. Thyroid 32:1158–1168. 10.1089/thy.2022.0324 35959734 10.1089/thy.2022.0324

[18] Perros P, Nagy EV, Papini E, Van Der Feltz-Cornelis C, Weetman AP, Hay HA et al (2023) Hypothyroidism and somatization: results from E-mode patient self-assessment of thyroid therapy, a cross-sectional, international online patient survey. Thyroid 33:927–939. 10.1089/thy.2022.0641 37134204 10.1089/thy.2022.0641

[19] Wiersinga WM, Duntas L, Fadeyev V, Nygaard B, Vanderpumpe MPJ (2012) 2012 ETA guidelines: the Use of L-T4 + L-T3 in the treatment of hypothyroidism. Eur Thyroid J 1:55–71. 10.1159/000339444 24782999 10.1159/000339444 PMC3821467

[20] Baloch Z, Carayon P, Conte-Devolx B, Demers LM, Feldt-Rasmussen U, Henry JF et al (2003) Laboratory medicine practice guidelines. Laboratory support for the diagnosis and monitoring of thyroid disease. Thyroid 13:3–126. 10.1089/105072503321086962 12625976 10.1089/105072503321086962

[21] Surks MI, Boucai L (2010) Age- and race-based serum thyrotropin reference limits. J Clin Endocrinol Metab 95:496–502. 10.1210/jc.2009-1845 19965925 10.1210/jc.2009-1845

[22] Surks MI (2013) TSH reference limits: new concepts and implications for diagnosis of subclinical hypothyroidism. Endocr Pract 19:1066–1069. 10.4158/EP13246.CO 24014005 10.4158/EP13246.CO

[23] Vadiveloo T, Donnan PT, Murphy MJ, Leese GP (2013) Age- and gender-specific TSH reference intervals in people with no obvious thyroid disease in Tayside, Scotland: the Thyroid Epidemiology, Audit, and Research Study (TEARS). J Clin Endocrinol Metab 98:1147–1153. 10.1210/jc.2012-3191 23345094 10.1210/jc.2012-3191

[24] Somwaru LL, Arnold AM, Joshi N, Fried LP, Cappola AR (2009) High frequency of and factors associated with thyroid hormone over-replacement and under-replacement in men and women aged 65 and over. J Clin Endocrinol Metab 94:1342–1345. 10.1210/jc.2008-1696 19126628 10.1210/jc.2008-1696 PMC2682480

[25] Wekking EM, Appelhof BC, Fliers E, Schene AH, Huyser J, Tijssen JGP, Wiersinga WM (2005) Cognitive functioning and well-being in euthyroid patients on thyroxine replacement therapy for primary hypothyroidism. Eur J Endocrinol 153:747–753. 10.1530/eje.1.02025 16322379 10.1530/eje.1.02025

[26] Klubo-Gwiezdzinska J, Wartofsky L (2022) Hashimoto thyroiditis: an evidence-based guide to etiology, diagnosis and treatment. Pol Arch Intern Med 132:16222. 10.20452/pamw.16222 35243857 10.20452/pamw.16222 PMC9478900

[27] Arafah BM (1994) Decreased levothyroxine requirement in women with hypothyroidism during androgen therapy for breast cancer. Ann Intern Med 121:247–251. 10.7326/0003-4819-121-4-199408150-00002 7518657 10.7326/0003-4819-121-4-199408150-00002

[28] Checchi S, Montanaro A, Pasqui L, Ciuoli C, de Palo V, Chiappetta MC, Pacini F (2008) L-thyroxine requirement in patients with autoimmune hypothyroidism and parietal cell antibodies. J Clin Endocrinol Metab 93:465–469. 10.1210/jc.2007-1544 18042648 10.1210/jc.2007-1544

[29] Virili C, Bassotti G, Santaguida MG, Iuorio R, Del Duca SC, Mercuri V et al (2012) Atypical celiac disease as cause of increased need for thyroxine: a systematic study. J Clin Endocrinol Metab 97:E419–E422. 10.1210/jc.2011-1851 22238404 10.1210/jc.2011-1851

[30] Sawin CT, Geller A, Wolf PA, Belanger AJ, Baker E, Bacharach P et al (1994) Low serum thyrotropin concentrations as a risk factor for atrial fibrillation in older persons. N Engl J Med 331:1249–1252. 10.1056/NEJM199411103311901 7935681 10.1056/NEJM199411103311901

[31] Flynn RW, Bonellie SR, Jung RT, MacDonald TM, Morris AM, Leese GP (2010) Serum thyroid-stimulating hormone concentration and morbidity from cardiovascular disease and fractures in patients on long-term thyroxine therapy. J Clin Endocrinol Metab 95:186–193. 10.1210/jc.2009-1625 19906785 10.1210/jc.2009-1625

[32] Lillevang-Johansen M, Abrahamsen B, Jørgensen HL, Brix TH, Hegedüs L (2019) Duration of over- and under-treatment of hypothyroidism is associated with increased cardiovascular risk. Eur J Endocrinol 180:407–416. 10.1530/EJE-19-0006 31035256 10.1530/EJE-19-0006

[33] Guglielmi R, Frasoldati A, Zini M, Grimaldi F, Gharib H, Garber JR, Papini E (2016) Italian Association of Clinical Endocrinologists statement Replacement therapy for primary hypothyroidism: a brief guide for clinical practice. Endocr Pract 22:1319–1326. 10.4158/EP161308.OR 27482609 10.4158/EP161308.OR

[34] Biondi B, Cooper DS (2019) Thyroid hormone therapy for hypothyroidism. Endocrine 66:18–26. 10.1007/s12020-019-02023-7 31372822 10.1007/s12020-019-02023-7

[35] Guyatt GH et al (2008) GRADE: an emerging consensus on rating quality of evidence and strength of recommendations. BMJ 336:924–926. 10.1136/bmj.39489.470347.AD 18436948 10.1136/bmj.39489.470347.AD PMC2335261

[36] Brouwers MC et al (2010) AGREE II: advancing guideline development, reporting and evaluation in health care. Prev Med 51:421–424. 10.1016/j.ypmed.2010.08.005 20728466 10.1016/j.ypmed.2010.08.005

[37] Shea BJ, Reeves BC, Wells G et al (2017) AMSTAR 2: a critical appraisal tool for systematic reviews that include randomised or non-randomised studies of healthcare interventions, or both. BMJ 358:j4008. 10.1136/bmj.j4008 28935701 10.1136/bmj.j4008 PMC5833365

[38] Higgins JPT, Altman DG, Gøtzsche PC et al (2011) The Cochrane Collaboration’s tool for assessing risk of bias in randomised trials. BMJ 343:d5928. 10.1136/bmj.d5928 22008217 10.1136/bmj.d5928 PMC3196245

[39] The GRADEPro Guideline Development Tool (GDT). (https://training.cochrane.org/resource/tsc-induction-mentoring-training-guide/7-archie-and-revman/88-gradepro).

[40] The Appraisal of Guidelines for REsearch & Evaluation. (https://www.agreetrust.org/wp-content/uploads/2016/02/AGREE-Reporting-Checklist-2016.pdf).

[41] Ruggeri M, Basile M, Armuzzi A, Cicchetti A (2016) Activity-based costing and budget analysis of vedolizumab versus conventional treatments in ulcerative colitis and Crohn’s disease. Global Reg Health Technol Assess 4:e88-99. 10.33393/grhta.2017.382

[42] Lan H, Wen J, Mao Y, Huang H, Chen G, Lin W (2022) Combined T4 + T3 therapy versus T4 monotherapy effect on psychological health in hypothyroidism: A systematic review and meta-analysis. Clin Endocrinol (Oxf) 97:13–25. 10.1111/cen.14742 35445422 10.1111/cen.14742

[43] Millan-Alanis JM, González-González JG, Flores-Rodríguez A, Singh Ospina N, Maraka S, Moreno-Peña PJ et al (2021) Benefits and harms of levothyroxine/l-triiodothyronine versus levothyroxine monotherapy for adult patients with hypothyroidism: systematic review and meta-analysis. Thyroid 31:1613–1625. 10.1089/thy.2021.0270 34340589 10.1089/thy.2021.0270 PMC8917901

[44] Appelhof BC, Fliers E, Wekking EM, Schene AH, Huyser J, Tijssen JG et al (2005) Combined therapy with levothyroxine and liothyronine in two ratios, compared with levothyroxine monotherapy in primary hypothyroidism: a double-blind, randomized, controlled clinical trial. J Clin Endocrinol Metab 90:2666–2674. 10.1219/jc.2004-2111 15705921 10.1210/jc.2004-2111

[45] Valizadeh M, Seyyed-Majidi MR, Hajibeigloo H, Momtazi S, Musavinasab N, Hayatbakhsh MR (2009) Efficacy of combined levothyroxine and liothyronine as compared with levothyroxine monotherapy in primary hypothyroidism: a randomized controlled trial. Endocr Res 34:80–89. 10.1080/07435800903156340 19701833 10.1080/07435800903156340

[46] Biondi B, Pucci M, Pontieri G, Formisano P, Esposito R (2023) Preliminary results of a double-blind randomized controlled trial evaluating the cardio-metabolic effects of levothyroxine and liothyronine compared to levothyroxine with placebo in athyreotic low risk thyroid cancer patients. Thyroid 33:1402–1413. 10.1089/thy.2023.0135 37725587 10.1089/thy.2023.0135

[47] Brigante G, Santi D, Boselli G, Margiotta G, Corleto R, Monzani ML et al (2024) Randomized double-blind placebo-controlled trial on levothyroxine and liothyronine combination therapy in totally thyroidectomized subjects: the LEVOLIO study. Eur J Endocrinol 190:12–22. 10.1093/ejendo/lvad172 38124252 10.1093/ejendo/lvad172

[48] Bjerkreim BA, Hammerstad SS, Gulseth HL, Berg TJ, Lee-Ødegård S, Eriksen EF (2022) Thyroid signaling biomarkers in female symptomatic hypothyroid patients on liothyronine versus levothyroxine monotherapy: a randomized crossover trial. J Thyroid Res 2022:6423023. 10.1155/2022/6423023 35572853 10.1155/2022/6423023 PMC9095395

[49] Bjerkreim BA, Hammerstad SS, Gulseth HL, Berg TJ, Omdal LJ, Lee-Ødegård S, Eriksen EF (2022) Effect of liothyronine treatment on quality of life in female hypothyroid patients with residual symptoms on levothyroxine therapy: a randomized crossover study. Front Endocrinol (Lausanne) 13:816566. 10.3389/fendo.2022.816566 35273566 10.3389/fendo.2022.816566 PMC8902821

[50] Fadeyev VV, Morgunova TB, Sytch JP, Melnichenko GA (2005) TSH and thyroid hormones concentrations in patients with hypothyroidism receiving replacement therapy with L-thyroxine alone or in combination with L-triiodothyronine. Hormones (Athens) 4:101–107 (**PMID: 16613812**) 16613812

[51] Fadeyev VV, Morgunova TB, Melnichenko GA, Dedov II (2010) Combined therapy with L-Thyroxine and L-Triiodothyronine compared to L-Thyroxine alone in the treatment of primary hypothyroidism. Hormones 9:245–252. 10.14310/horm.2002.1274 20688622 10.14310/horm.2002.1274

[52] Saravanan P, Simmons DJ, Greenwood R, Peters TJ, Dayan CM (2005) Partial substitution of thyroxine (T4) with tri-iodothyronine in patients on T4 replacement therapy: results of a large community-based randomized controlled trial. J Clin Endocrinol Metab 90:805–812. 10.1210/jc.2004-1672 15585551 10.1210/jc.2004-1672

[53] Celi FS, Zemskova M, Linderman JD, Smith S, Drinkard B, Sachdev V et al (2011) Metabolic effects of liothyronine therapy in hypothyroidism: a randomized, double-blind, crossover trial of liothyronine versus levothyroxine. J Clin Endocrinol Metab 96:3466–3474. 10.1210/jc.2011-1329 21865366 10.1210/jc.2011-1329 PMC3205882

[54] Celi FS, Zemskova M, Linderman JD, Babar NI, Skarulis MC, Csako G et al (2010) The pharmacodynamic equivalence of levothyroxine and liothyronine: a randomized, double blind, cross-over study in thyroidectomized patients. Clin Endocrinol 72:709–715. 10.1111/j.1365-2265.2009.03700.x 10.1111/j.1365-2265.2009.03700.x PMC2888764 20447070

[55] Clyde PW, Harari AE, Getka EJ, Shakir KM (2003) Combined levothyroxine plus liothyronine compared with levothyroxine alone in primary hypothyroidism: a randomized controlled trial. JAMA 290:2952–2958. 10.1001/jama.290.22.2952 14665656 10.1001/jama.290.22.2952

[56] Hoang TD, Olsen CH, Mai VQ, Clyde PW, Shakir MK (2013) Desiccated thyroid extract compared with levothyroxine in the treatment of hypothyroidism: a randomized, double-blind, crossover study. J Clin Endocrinol Metab 98:1982–1990. 10.1210/jc.2012-4107 23539727 10.1210/jc.2012-4107

[57] Rodriguez T, Lavis VR, Meininger JC, Kapadia AS, Stafford LF (2005) Substitution of liothyronine at a 1: 5 ratio for a portion of levothyroxine: effect on fatigue, symptoms of depression, and working memory versus treatment with levothyroxine alone. Endocr Pract 11:223–233. 10.4158/EP.11.4.223 16006298 10.4158/EP.11.4.223 PMC1455482

[58] Sawka AM, Gerstein HC, Marriott MJ, MacQueen GM, Joffe RT (2003) Does a combination regimen of thyroxine (T4) and 3,5,3’-triiodothyronine improve depressive symptoms better than T4 alone in patients with hypothyroidism? Results of a double-blind, randomized, controlled trial. J Clin Endocrinol Metab 88:4551–4555. 10.1210/jc.2003-030139 14557420 10.1210/jc.2003-030139

[59] Shakir MKM, Brooks DI, McAninch EA, Fonseca TL, Mai VQ, Bianco AC, Hoang TD (2021) Comparative effectiveness of levothyroxine, desiccated thyroid extract, and levothyroxine + liothyronine in hypothyroidism. J Clin Endocrinol Metab 106:e4400–e4413. 10.1210/clinem/dgab478 34185829 10.1210/clinem/dgab478 PMC8530721

[60] Pierannunzio D et al (2022) Thyroidectomies in Italy: A population-based national analysis from 2001 to 2018. Thyroid 32:263–272. 10.1089/thy.2021.0531 35018816 10.1089/thy.2021.0531

[61] Cappelli C, Pirola I, Daffini L, Formenti A, Iacobello C, Cristiano A et al (2016) A double-blind placebo-controlled trial of liquid thyroxine ingested at breakfast: results of the TICO study. Thyroid 26:197–202. 10.1089/thy.2015.0422 26586610 10.1089/thy.2015.0422

[62] Guglielmi R, Grimaldi F, Negro R, Frasoldati A, Misischi I, Graziano F et al (2018) Shift from levothyroxine tablets to liquid formulation at breakfast improves quality of life of hypothyroid patients. Endocr Metab Immune Disord Drug Targets 18:235–240. 10.2174/1871530318666180125155348 29376496 10.2174/1871530318666180125155348 PMC5997842

[63] Benvenga S, Bartolone L, Squadrito S, Lo Giudice F, Trimarchi F (1995) Delayed intestinal absorption of levothyroxine. Thyroid 5:249–253. 10.1089/thy.1995.5.249 7488863 10.1089/thy.1995.5.249

[64] Virili C, Bruno G, Santaguida MG, Gargano L, Stramazzo I, De Vito C et al (2022) Levothyroxine treatment and gastric juice pH in humans: the proof of concept. Endocrine 77:102–111. 10.1007/s12020-022-03056-1 35477833 10.1007/s12020-022-03056-1 PMC9242941

[65] Virili C, Brusca N, Capriello S, Centanni M (2021) Levothyroxine therapy in gastric malabsorptive disorders. Front Endocrinol (Lausanne) 11:621616. 10.3389/fendo.2020.621616 33584549 10.3389/fendo.2020.621616 PMC7876372

[66] Liwanpo L, Hershman JM (2009) Conditions and drugs interfering with thyroxine absorption. Best Pract Res Clin Endocrinol Metab 23:781–792. 10.1016/j.beem.2009.06.006 19942153 10.1016/j.beem.2009.06.006

[67] McMillan M, Rotenberg KS, Vora K, Sterman AB, Thevathasan L, Ryan MF et al (2016) Comorbidities, concomitant medications, and diet as factors affecting levothyroxine therapy: results of the CONTROL surveillance project. Drugs R D 16:53–68. 10.1007/s40268-015-0116-6 26689565 10.1007/s40268-015-0116-6 PMC4767717

[68] Caron P, Grunenwald S, Persani L, Borson-Chazot F, Leroy R, Duntas L (2022) Factors influencing the levothyroxine dose in the hormone replacement therapy of primary hypothyroidism in adults. Rev Endocr Metab Disord 23:463–483. 10.1007/s11154-021-09691-9 34671932 10.1007/s11154-021-09691-9 PMC8528480

[69] Wiesner A, Gajewska D, Paśko P (2021) Levothyroxine interactions with food and dietary supplements - A systematic review. Pharmaceuticals (Basel) 14:206. 10.3390/ph14030206 33801406 10.3390/ph14030206 PMC8002057

[70] Liu H, Lu M, Hu J, Fu G, Feng Q, Sun S, Chen C (2023) Medications and food interfering with the bioavailability of levothyroxine: a systematic review. Ther Clin Risk Manag 19:503–523. 10.2147/TCRM.S414460 37384019 10.2147/TCRM.S414460 PMC10295503

[71] Skelin M, Lucijanić T, Liberati-Čizmek AM, Klobučar SM, Lucijanić M, Jakupović L et al (2018) Effect of timing of levothyroxine administration on the treatment of hypothyroidism: a three-period crossover randomized study. Endocrine 62:432–439. 10.1007/s12020-018-1686-1 30043093 10.1007/s12020-018-1686-1

[72] de Mello RB, Giassi K, Stahl G, Machado Assis ML, Flores MS, de Lima BC et al (2022) Evaluation of bedtime vs morning levothyroxine intake to control hypothyroidism in older patients: a pragmatic crossover randomized clinical trial. Front Med (Lausanne) 9:828762. 10.3389/fmed.2022.828762 35814782 10.3389/fmed.2022.828762 PMC9261378

[73] Bolk N, Visser TJ, Nijman J, Jongste IJ, Tijssen JG, Berghout A (2010) Effects of evening vs morning levothyroxine intake: a randomized double-blind crossover trial. Arch Intern Med 170:1996–2003. 10.1001/archinternmed.2010.436 21149757 10.1001/archinternmed.2010.436

[74] Burch HB, Burman KD, Cooper DS, Hennessey JV (2014) A 2013 survey of clinical practice patterns in the management of primary hypothyroidism. J Clin Endocrinol Metab 99:2077–2085. 10.1210/jc.2014-1046 24527720 10.1210/jc.2014-1046

[75] Trimboli P, Scappaticcio L, De Bellis A, Maiorino MI, Knappe L, Esposito K et al (2020) Different formulations of levothyroxine for treating hypothyroidism: a real-life study. Int J Endocrinol 2020:4524759. 10.1155/2020/4524759 32184819 10.1155/2020/4524759 PMC7059087

[76] Jonklaas J, Burman KD (2016) Daily administration of short-acting liothyronine is associated with significant triiodothyronine excursions and fails to alter thyroid-responsive parameters. Thyroid 26:770–778. 10.1089/thy.2015.0629 27030088 10.1089/thy.2015.0629 PMC4913511

[77] Laurberg P, Andersen S, Bülow Pedersen I, Carlé A (2005) Hypothyroidism in the elderly: pathophysiology, diagnosis and treatment. Drugs Aging 22:23–38. 10.2165/00002512-200522010-00002 15663347 10.2165/00002512-200522010-00002

[78] Andersen S, Pedersen KM, Bruun NH, Laurberg P (2002) Narrow individual variations in serum T(4) and T(3) in normal subjects: a clue to the understanding of subclinical thyroid disease. J Clin Endocrinol Metab 87:1068–1072. 10.1210/jcem.87.3.8165 11889165 10.1210/jcem.87.3.8165

[79] Mohammadi K, Shafie D, Vakhshoori M, Bondariyan N, Rezvanian H, Heidarpour M (2023) Prevalence of amiodarone-induced hypothyroidism. a systematic review and meta-analysis. Trends Cardiovasc Med 33:252–262. 10.1016/j.tcm.2022.01.001 35026394 10.1016/j.tcm.2022.01.001

[80] Danzi S, Klein I (2015) Amiodarone-induced thyroid dysfunction. J Intensive Care Med 30:179–185. 10.1177/0885066613503278 24067547 10.1177/0885066613503278

[81] Fairbrother F, Petzl N, Scott JG, Kisely S (2019) Lithium can cause hyperthyroidism as well as hypothyroidism: a systematic review of an under-recognised association. Aust N Z J Psychiatry 53:384–402. 10.1177/0004867419833171 30841715 10.1177/0004867419833171

[82] Bohnet HG (2020) Monitoring of thyroid malfunction and therapies in pregnancy and the postpartum period: a systematic updated critical review of the literature. Ther Drug Monit 42:222–228. 10.1097/FTD.0000000000000691 31425445 10.1097/FTD.0000000000000691

[83] Okosieme OE, Khan I, Taylor PN (2018) Preconception management of thyroid dysfunction. Clin Endocrinol (Oxf) 89:269–279. 10.1111/cen.13731 29706030 10.1111/cen.13731

[84] Hamza A, Schlembach D, Schild RL, Groten T, Wölfie J, Battefeld W et al (2023) Recommendations of the AGG (Working Group for Obstetrics, Department of Maternal Diseases) on how to treat thyroid function disorders in pregnancy. Geburtshilfe Frauenheilkd 83:504–516. 10.1055/a-1967-1653 37152543 10.1055/a-1967-1653 PMC10159725

[85] Bensenor IM, Olmos RD, Lotufo PA (2012) Hypothyroidism in the elderly: diagnosis and management. Clin Interv Aging 7:97–111. 10.2147/CIA.S23966 22573936 10.2147/CIA.S23966 PMC3340110

[86] Duntas LH, Yen PM (2019) Diagnosis and treatment of hypothyroidism in the elderly. Endocrine 66:63–69. 10.1007/s12020-019-02067-9 31482381 10.1007/s12020-019-02067-9

[87] Feller M, Snel M, Moutzouri E, Bauer DC, De Montmollin M, Aujesky D et al (2018) Association of thyroid hormone therapy with quality of life and thyroid-related symptoms in patients with subclinical hypothyroidism: a systematic review and meta-analysis. JAMA 320:1349–1359. 10.1001/jama.2018.13770 30285179 10.1001/jama.2018.13770 PMC6233842

[88] Sawka AM, Cappola AR, Peeters RP, Kopp PA, Bianco AC, Jonklaas J (2019) Patient context and thyrotropin levels are important when considering treatment of subclinical hypothyroidism. Thyroid 29:1359–1363. 10.1089/thy.2019.0494 31489828 10.1089/thy.2019.0494

[89] Vezzani S et al (2018) An Italian survey of compliance with major guidelines for L-thyroxine of primary hypothyroidism. Endocr Pract 24:419–428. 10.4158/EP-2017-0159 29847168 10.4158/EP-2017-0159

[90] Lum SM, Nicoloff JT, Spencer CA, Kaptein EM (1984) Peripheral tissue mechanism for maintenance of serum triiodothyronine values in a thyroxine-deficient state in man. J Clin Invest 73:570–575. 10.1172/JCI111245 6699177 10.1172/JCI111245 PMC425050

[91] Basaria S, Cooper DS (2005) Amiodarone and the thyroid. Am J Med 118:706–714. 10.1016/j.amjmed.2004.11.028 15989900 10.1016/j.amjmed.2004.11.028

[92] Stockigt JR (2001) Free thyroid hormone measurement. a critical appraisal. Endocrinol Metab Clin North Am 30:265–289. 10.1016/s0889-8529(05)70187-0 11444163 10.1016/s0889-8529(05)70187-0

[93] Ain KB, Pucino F, Shiver TM, Banks SM (1993) Thyroid hormone levels affected by time of blood sampling in thyroxine-treated patients. Thyroid 3:81–85. 10.1089/thy.1993.3.81 8369656 10.1089/thy.1993.3.81

[94] Kaplan MM, Larsen PR, Crantz FR, Dzau VJ, Rossing TH, Haddow JE (1982) Prevalence of abnormal thyroid function test results in patients with acute medical illnesses. Am J Med 72:9–16. 10.1016/0002-9343(82)90565-4 6800256 10.1016/0002-9343(82)90565-4

[95] Peeters RP, Wouters PJ, Kaptein E, van Toor H, Visser TJ, Van den Berghe G (2003) Reduced activation and increased inactivation of thyroid hormone in tissues of critically ill patients. J Clin Endocrinol Metab 88:3202–3211. 10.1210/jc.2002-022013 12843166 10.1210/jc.2002-022013

[96] Taylor PN, Iqbal A, Minassian C, Sayers A, Draman MS, Greenwood R et al (2014) Falling threshold for treatment of borderline elevated thyrotropin levels-balancing benefits and risks: evidence from a large community-based study. JAMA Intern Med 174:32–39. 10.1001/jamainternmed.2013.11312 24100714 10.1001/jamainternmed.2013.11312

[97] Cappola AR (2014) Levothyroxine prescription: not as simple as it seems. JAMA 311:2532–2353. 10.1001/jama.2014.3808 25058086 10.1001/jama.2014.3808

[98] Vaidya B, Pearce SH (2008) Management of hypothyroidism in adults. BMJ 337:a801. 10.1136/bmj.a801 18662921 10.1136/bmj.a801

[99] Eligar V, Taylor PN, Okosieme OE, Leese GP, Dayan CM (2016) Thyroxine replacement: a clinical endocrinologist’s viewpoint. Ann Clin Biochem 53:421–433. 10.1177/0004563216642255 27126268 10.1177/0004563216642255

[100] Garber JR, Cobin RH, Gharib H, Hennessey JV, Klein I, Mechanick JI, American Association of Clinical Endocrinologists and American Thyroid Association taskforce on hypothyroidism in adults et al (2012) Clinical practice guidelines for hypothyroidism in adults: cosponsored by the American Association of Clinical Endocrinologists and the American Thyroid Association. Thyroid 22:1200–1235. 10.1089/thy.2012.0205 22954017 10.1089/thy.2012.0205

[101] Attanasio R, Žarković M, Papini E, Nagy EV, Negro R, Perros P et al (2024) Patients’ persistent symptoms, clinician demographics, and geo-economic factors are associated with choice of therapy for hypothyroidism by European thyroid specialists: the “THESIS” collaboration. Thyroid 34:429–441. 10.1089/thy.2023.0580 38368541 10.1089/thy.2023.0580

[102] Aghini Lombardi F et al (2013) The effect of voluntary iodine prophylaxis in a small rural community: the Pescopagano survey 15 years later. J Clin Endocrinol Metab 98:1031–1039. 10.1210/jc.2012-2960 23436921 10.1210/jc.2012-2960

[103] Italian Thyroid Cancer Observatory (ITCO). https://www.firstitco.org.

[104] Cooper D, Kaur R, Ayeni FE, Eslick GD, Edirimanne S (2024) Hypothyroidism after hemithyroidectomy: a systematic review and meta-analysis. Thyroid Res 17:18. 10.1186/s13044-024-00200-z 38972987 10.1186/s13044-024-00200-z PMC11229296

[105] Spandonaro F. L'aderenza nella governance della long-term care: proposta di indicatore sintetico. Centro per la Ricerca Economica Applicata in Sanità 2020. Scheda di sintesi dell’Expert Opinion Paper di Italia Longeva.

[106] Biondi B, Cooper DS (2008) The clinical significance of subclinical thyroid dysfunction. Endocr Rev 29:76–131. 10.1210/er.2006-0043 17991805 10.1210/er.2006-0043

[107] Hennessey JV (2017) The emergence of levothyroxine as a treatment for hypothyroidism. Endocrine 55:6–18. 10.1007/s12020-016-1199-8 27981511 10.1007/s12020-016-1199-8

[108] Penna GC, Salas-Lucia F, Ribeiro MO, Bianco AC (2024) Gene polymorphisms and thyroid hormone signaling: implication for the treatment of hypothyroidism. Endocrine 84(2):309–319. 10.1007/s12020-023-03528-y 37740833 10.1007/s12020-023-03528-y PMC10959761

