# Supplementary material for: Italian guidelines for the management of adult individuals with primary hypothyroidism outside pregnancy
Source: J Endocrinol Invest. 2025 Jul 23;48(10):2295–313. doi: 10.1007/s40618-025-02652-y (PMC12518491; doi:10.1007/s40618-025-02652-y)
Supplement: Supplementary file 1 — Supplementary file1 (DOCX 138 KB) [file 40618_2025_2652_MOESM1_ESM.docx]

**7. SUPPLEMENTARY MATERIAL**

**Appendix 1**

**Guideline Development Team**

**Chair**: Rinaldo Guglielmi (endocrinologist)

**Panel members**:

- Rinaldo Guglielmi (endocrinologist)
- Caterina Mian (endocrinologist)
- Claudio Andreoli (general practitioner, endocrinologist)
- Marcello Bagnasco (endocrinologist)
- Marco Boniardi (surgeon)
- Ernesto De Menis (internal medicine)
- Maria Luisa De Rimini (nuclear medicine)
- Tina Doris (nurse)
- Giulia Fiorentini (psychologist)
- Pietro Locantore (endocrinologist)
- Simone Mauramati (ENT surgeon)
- Enrico Papini (endocrinologist)
- Federica Presciuttini (endocrinologist, representative of patients)
- Marco Raffaelli (surgeon)
- Silvia Rizzati (endocrinologist, internal medicine)
- Maria Gabriella Rugiu (ENT surgeon)
- Marsida Teliti (endocrinologist)
- Vincenzo Triggiani (endocrinologist)
- Annibale Versari (nuclear medicine)
- Camilla Virili (endocrinologist)
- Marina Vitillo (laboratory medicine)

**Evidence Review Team**:

- Michele Basile, Fabio Cruciani, Zuzana Mitrova, Rosella Saulle, Ilaria Valentini

**External reviewers**:

- Mirco Bartolomei (nuclear medicine)
- Giulia Bertino (ENT surgeon)
- Anna Maria Biancifiori (representative of patients)
- Pietro Giorgio Calò (surgeon)
- Federica D’Aurizio (laboratory medicine)
- Caterina Di Cosmo (endocrinologist)
- Andrea Frasoldati (endocrinologist)
- Massimo Marchetti (internal medicine, endocrinologist)
- Rosa Elisa Miceli (psychologist)
- Salvatore Monti (endocrinologist)
- Alfredo Pontecorvi (endocrinologist)
- Mario Rotondi (endocrinologist)

**AME guideline team**

- Alessandro Scoppola, team coordinator (endocrinologist)
- Roberto Attanasio, Roberto Novizio, Agostino Paoletta, Agnese Persichetti, Irene Samperi (endocrinologists)

**Appendix 2**

**Clinical question and inclusion criteria (PICO)**

**Clinical question:** What is the most effective and safest treatment for permanent overt primary hypothyroidism **(**autoimmune hypothyroidism or iatrogenic hypothyroidism due to surgery, radiodine, or drugs, such as amiodarone or lithium)?

**Population**: The population of interest includes adults (>18 years old) with primary hypothyroidism.

**Exclusion criteria**: pregnant, puerperal and breastfeeding women; subjects with central hypothyroidism; subjects with thyroid carcinoma still requiring suppressive therapy; subjects with transient iatrogenic hypothyroidism (immuno-oncological drugs, etc.).

**Intervention:** treatment with oral levothyroxine (LT4) (in any formulation).

**Comparisons:**

- LT3 therapy
- LT4 + LT3 combination therapy
- thyroid extract therapy

**Outcomes**

- Reduction of symptoms: asthenia, cold intolerance, constipation, depression, ideomotor slowing, myalgia, lethargy
- Reduction of signs: heart rate variation, edema, dry skin, hair fragility, weight gain, myxedema, hoarse voice
- Achievement of normal hormone values (TSH, FT4) according to age and comorbidity
- Change in lipid profile: total, HDL, and LDL cholesterol
- Treatment complications and side effects: subclinical/clinical thyrotoxicosis
- Quality of life
- Adherence
- Death

**Design of searched studies:** Systematic reviews of randomized controlled trials (RCTs) and observational studies. If not available, RCTs observational studies were searched. In case of multiple relevant reviews for the same clinical question, the review with the highest methodological quality based on the results of the evaluation conducted with the AMSTAR 2 checklist was selected.

## **Appendix 3**

## **Search strategy**

**EFFICACY**

**Search for systematic reviews (until December 18, 2023)**

**Cochrane *Library* (*issue* 12, 2023)**

#1 MeSH descriptor: [Hypothyroidism] explode all trees

#2 hypothyr*:ti,ab

#3 hypo-thyr*:ti,ab,kw

#4 (thyroid* NEAR/3 (defic* OR insuffic* OR failure)):ti,ab,kw

#5 (TSH NEAR/2 Deficien*):ti,ab,kw

#6 (thyroid* low product*):ti,ab,kw

#7 (thyroid* under product*):ti,ab,kw

#8 (thyroid* underactive*):ti,ab,kw

#9 {OR #1-#8}

#10 MeSH descriptor: [Thyroxine] explode all trees

#11 (levothyrox* OR laevothyrox* OR levo-thyrox* OR laevo-thyrox* OR l-thyrox* OR levo-t OR L-T4 OR LT4):ti,ab,kw

#12 (berlthyrox OR dexnon OR eferox OR eltroxin OR eltroxine OR euthyrox OR eutirox OR "l thyrox" OR "l thyroxin henning" OR "l thyroxin beta" OR tetraiodothyronine OR "levo t" OR levothroid OR levothyroid OR levoxine OR levoxyl OR novothyral OR novothyrox OR oroxine OR q51bo43mg4 OR synthroid OR synthrox OR "t4 thyroid hormone" OR thevier OR thyrax OR "thyroid hormone, t4" OR thyroxin OR thyroxine OR tiroidine OR "tiroxina leo" OR unithroid):ti,ab,kw

#13 (Thyroid substitution therapy):ti,ab,kw

#14 {OR #10-#13}

#15 #9 AND #14 in Cochrane Reviews

***Database*: Ovid MEDLINE(R) ALL <since 1946 to December 15, 2023>**

**Search Strategy:**

**1** exp Hypothyroidism/

**2** hypothyr*.mp.

**3** hypo-thyr*.mp.

**4** (thyroid? adj3 (defic* or insuffic* or failure?)).mp.

**5** (thyroid? adj3 low adj3 product*).mp.

**6** (thyroid? adj3 under adj3 product*).mp.

**7** (thyroid? adj3 underactive*).mp.

**8** (tsh adj3 defic*).mp.

**9** 1 or 2 or 3 or 4 or 5 or 6 or 7 or 8

**10** Thyroxine/

**11** (levothyrox* or laevothyrox* or levo-thyrox* or laevo-thyrox* or l-thyrox* or levo-t or L-T4 or LT4).mp.

**12** (3,5,3',5'-tetraiodothyronine or 51-48-9 or "7488-70-2 (thyroxine)" or berlthyrox or dexnon or eferox or eltroxin or eltroxine or euthyrox or eutirox or l thyrox or l thyroxin henning or l thyroxin beta or l thyroxine or l thyroxine roche or l-3,5,3',5'-tetraiodothyronine or levo t or levothroid or levothyroid or levoxine or levoxyl or novothyral or novothyrox or "o-(4-hydroxy-3,5-diiodophenyl) 3,5-diiodo-l-tyrosine" or "o-(4-hydroxy-3,5-diiodophenyl)-3,5-diiodotyrosine" or oroxine or q51bo43mg4 or synthroid or synthrox or t4 thyroid hormone or thevier or thyrax or thyroid hormone, t4 or thyroxin or thyroxine or tiroidine or tiroxina leo or unithroid).ti,ab.

**13** thyroid substitution therapy.mp.

**14** 10 or 11 or 12 or 13

**15** 9 and 14

**16** meta-analysis/ or systematic review/ or meta-analysis as topic/ or "meta analysis (topic)"/ or "systematic review (topic)"/ or exp technology assessment, biomedical/

**17** ((systematic* adj3 (review* or overview*)) or (methodologic* adj3 (review* or overview*))).ti,ab,kf,kw.

**18** ((quantitative adj3 (review* or overview* or synthes*)) or (research adj3 (integrati* or overview*))).ti,ab,kf,kw.

**19** ((integrative adj3 (review* or overview*)) or (collaborative adj3 (review* or overview*)) or (pool* adj3 analy*)).ti,ab,kf,kw.

**20** (data synthes* or data extraction* or data abstraction*).ti,ab,kf,kw.

**21** (handsearch* or hand search*).ti,ab,kf,kw.

**22** (handsearch* or hand search*).ti,ab,kf,kw.

**23** (meta regression* or metaregression*).ti,ab,kf,kw.

**24** (meta-analy* or metaanaly* or systematic review* or biomedical technology assessment* or bio-medical technology assessment*).mp,hw.

**25** (medline or cochrane or pubmed or medlars or embase or cinahl).ti,ab,hw.

**26** (cochrane or (health adj2 technology assessment) or evidence report).jw.

**27** (comparative adj3 (efficacy or effectiveness)).ti,ab,kf,kw.

**28** (outcomes research or relative effectiveness).ti,ab,kf,kw.

**29** ((indirect or indirect treatment or mixed-treatment) adj comparison*).ti,ab,kf,kw.

**30** 16 or 17 or 18 or 19 or 20 or 21 or 22 or 23 or 24 or 25 or 26 or 27 or 28 or 29

**31** 15 and 30

***Database*: Embase <since 1974 to December 15, 2023>**

**Search strategy:**

**1** exp Hypothyroidism/

**2** hypothyr*.mp.

**3** hypo-thyr*.mp.

**4** (thyroid? adj3 (defic* or insuffic* or failure?)).mp.

**5** (thyroid? adj3 low adj3 product*).mp.

**6** (thyroid? adj3 under adj3 product*).mp.

**7** (thyroid? adj3 underactive*).mp.

**8** (tsh adj3 defic*).mp.

**9** 1 or 2 or 3 or 4 or 5 or 6 or 7 or 8

**10** levothyroxine/

**11** (levothyrox* or laevothyrox* or levo-thyrox* or laevo-thyrox* or l-thyrox* or levo-t or L-T4 or LT4).mp.

**12** ("3,5,3',5'-tetraiodothyronine" or "51-48-9" or "7488-70-2 (thyroxine)" or berlthyrox or dexnon or eferox or eltroxin or eltroxine or euthyrox or eutirox or l thyrox or l thyroxin henning or l thyroxin beta or l thyroxine or l thyroxine roche or "l-3,5,3',5'-tetraiodothyronine" or levo t or levothroid or levothyroid or levoxine or levoxyl or novothyral or novothyrox or "o-(4-hydroxy-3,5-diiodophenyl) 3,5-diiodo-l-tyrosine" or "o-(4-hydroxy-3,5-diiodophenyl)-3,5-diiodotyrosine" or oroxine or q51bo43mg4 or synthroid or synthrox or t4 thyroid hormone or thevier or thyrax or "thyroid hormone, t4" or thyroxin or thyroxine or tiroidine or tiroxina leo or unithroid).ti,ab.

**13** thyroid substitution therapy.mp.

**14** 10 or 11 or 12 or 13

**15** 9 and 14

**16** "systematic review"/ or meta analysis/

**17** "meta analysis (topic)"/

**18** "systematic review (topic)"/

**19** biomedical technology assessment/

**20** ((systematic* adj3 (review* or overview*)) or (methodologic* adj3 (review* or overview*))).ti,ab.

**21** ((quantitative adj3 (review* or overview* or synthes*)) or (research adj3 (integrati* or overview*))).ti,ab.

**22** ((integrative adj3 (review* or overview*)) or (collaborative adj3 (review* or overview*)) or (pool* adj3 analy*)).ti,ab.

**23** (data synthes* or data extraction* or data abstraction*).ti,ab.

**24** (handsearch* or hand search*).ti,ab.

**25** (mantel haenszel or peto or der simonian or dersimonian or fixed effect* or latin square*).ti,ab.

**26** (met analy* or metanaly* or technology assessment* or HTA or HTAs or technology overview* or technology appraisal*).ti,ab.

**27** (meta regression* or metaregression*).ti,ab.

**28** (meta-analy* or metaanaly* or systematic review* or biomedical technology assessment* or bio-medical technology assessment*).mp,hw.

**29** (medline or cochrane or pubmed or medlars or embase or cinahl).ti,ab.

**30** (cochrane or (health adj2 technology assessment) or evidence report).jw.

**31** (comparative adj3 (efficacy or effectiveness)).ti,ab.

**32** (outcomes research or relative effectiveness).ti,ab.

**33** ((indirect or indirect treatment or mixed-treatment) adj comparison*).ti,ab.

**34** 16 or 17 or 18 or 19 or 20 or 21 or 22 or 23 or 24 or 25 or 26 or 27 or 28 or 29 or 30 or 31 or 32 or 33

**35** 15 and 34

***Database*: APA PsycInfo <since 1806 to the first week of December 2023>**
**Search strategy:**

**1** exp Hypothyroidism/

**2** hypothyr*.tw.

**3** hypo-thyr*.tw.

**4** (thyroid? adj3 (defic* or insuffic* or failure?)).tw.

**5** (thyroid? adj3 low adj3 product*).tw.

**6** (thyroid? adj3 under adj3 product*).tw.

**7** (thyroid? adj3 underactive*).tw.

**8** (tsh adj3 defic*).tw.

**9** 1 or 2 or 3 or 4 or 5 or 6 or 7 or 8

**10** thyroxine/

**11** (levothyrox* or laevothyrox* or levo-thyrox* or laevo-thyrox* or l-thyrox* or levo-t or L-T4 or LT4).tw.

**12** (3,5,3',5'-tetraiodothyronine or 51-48-9 or "7488-70-2 (thyroxine)" or berlthyrox or dexnon or eferox or eltroxin or eltroxine or euthyrox or eutirox or l thyrox or l thyroxin henning or l thyroxin beta or l thyroxine or l thyroxine roche or l-3,5,3',5'-tetraiodothyronine or levo t or levothroid or levothyroid or levoxine or levoxyl or novothyral or novothyrox or "o-(4-hydroxy-3,5-diiodophenyl) 3,5-diiodo-l-tyrosine" or "o-(4-hydroxy-3,5-diiodophenyl)-3,5-diiodotyrosine" or oroxine or q51bo43mg4 or synthroid or synthrox or t4 thyroid hormone or thevier or thyrax or thyroid hormone, t4 or thyroxin or thyroxine or tiroidine or tiroxina leo or unithroid).tw.

**13** thyroid substitution therapy.tw.

**14** 10 or 11 or 12 or 13

**15** 9 and 14

**16** "systematic review"/ or meta analysis/

**17** ((systematic* adj3 (review* or overview*)) or (methodologic* adj3 (review* or overview*))).ti,ab.

**18** ((quantitative adj3 (review* or overview* or synthes*)) or (research adj3 (integrati* or overview*))).ti,ab.

**19** ((integrative adj3 (review* or overview*)) or (collaborative adj3 (review* or overview*)) or (pool* adj3 analy*)).ti,ab.

**20** (data synthes* or data extraction* or data abstraction*).ti,ab.

**21** (handsearch* or hand search*).ti,ab.

**22** (mantel haenszel or peto or der simonian or dersimonian or fixed effect* or latin square*).ti,ab.

**23** (met analy* or metanaly* or technology assessment* or HTA or HTAs or technology overview* or technology appraisal*).ti,ab.

**24** (meta regression* or metaregression*).ti,ab.

**25** (meta-analy* or metaanaly* or systematic review* or biomedical technology assessment* or bio-medical technology assessment*).mp,hw.

**26** (medline or cochrane or pubmed or medlars or embase or cinahl).ti,ab.

**27** (comparative adj3 (efficacy or effectiveness)).ti,ab.

**28** (outcomes research or relative effectiveness).ti,ab.

**29** ((indirect or indirect treatment or mixed-treatment) adj comparison*).ti,ab.

**30** 16 or 17 or 18 or 19 or 20 or 21 or 22 or 23 or 24 or 25 or 26 or 27 or 28 or 29

**31** 15 and 30

***Web of Science Core Collection***

- WOS.SCI: since 1980 to 2023

- WOS.AHCI: since 1980 to 2023

- WOS.ESCI: since 2018 to 2023

- WOS.ISTP: since 1990 to 2023

- WOS.SSCI: since 1980 to 2023

- WOS.ISSHP: since 1990 to 2023

# Searches:

1: TS=(hypothyr*) OR TS=(hypo-thyr*) OR TS=(thyroid$ NEAR/3 (defic* OR insuffic* OR failure$)) OR TS=(thyroid$ NEAR/3 low NEAR/3 product*) OR TS=(thyroid$ NEAR/3 under NEAR/3 product*) OR TS=(thyroid$ NEAR/3 underactive*) OR TS=(tsh NEAR/3 defic*) Date Run: Mon Dec 18 2023 12:44:50 GMT+0100 (Ora standard dell’Europa centrale)

2: (TS=(levothyrox* OR laevothyrox* OR levo-thyrox* OR laevo-thyrox* OR l-thyrox* OR levo-t OR L-T4 OR LT4)) OR TS=((berlthyrox OR dexnon OR eferox OR eltroxin OR eltroxine OR euthyrox OR eutirox OR "l thyrox" OR "l thyroxin henning" OR "l thyroxin beta" OR tetraiodothyronine OR "levo t" OR levothroid OR levothyroid OR levoxine OR levoxyl OR novothyral OR novothyrox OR oroxine OR q51bo43mg4 OR synthroid OR synthrox OR "t4 thyroid hormone" OR thevier OR thyrax OR "thyroid hormone, t4" OR thyroxin OR thyroxine OR tiroidine OR "tiroxina leo" OR unithroid)) Date Run: Mon Dec 18 2023 12:47:00 GMT+0100 (Ora standard dell’Europa centrale)

3: TS=((systematic* NEAR/3 (review* OR overview*)) OR (methodologic* NEAR/3 (review* OR overview*))) OR TS=((quantitative NEAR/3 (review* OR overview* OR synthes*)) OR (research NEAR/3 (integrati* OR overview*))) OR TS=((integrative NEAR/3 (review* OR overview*)) OR (collaborative NEAR/3 (review* OR overview*)) OR (pool* NEAR/3 analy*)) OR TS=("data synthes*" OR "data extraction*" OR "data abstraction*") OR TS=("met analy*" OR metanaly* OR "technology assessment*" OR HTA OR HTAs OR "technology overview*" OR "technology appraisal*") Date Run: Mon Dec 18 2023 12:47:52 GMT+0100 (Ora standard dell’Europa centrale)

4: #3 AND #2 AND #1 Date Run: Mon Dec 18 2023 12:48:14 GMT+0100 (Ora standard dell’Europa centrale)

**Epistemonikos**

Full query: (title:((levothyrox* OR laevothyrox* OR levo-thyrox* OR laevo-thyrox* OR l-thyrox* OR levo-t OR L-T4 OR LT4) AND (hypothyr* OR hypo-thyr*)) OR abstract:((levothyrox* OR laevothyrox* OR levo-thyrox* OR laevo-thyrox* OR l-thyrox* OR levo-t OR L-T4 OR LT4) AND (hypothyr* OR hypo-thyr*))) [Filters: classification=systematic-review, protocol=no]

**Search for RCTs until January 26, 2024**

***Cochrane Central Register of Controlled Trials***

**Issue 1, December 2023**

#1 MeSH descriptor: [Hypothyroidism] explode all trees

#2 hypothyr*:ti,ab

#3 hypo-thyr*:ti,ab,kw

#4 (thyroid* NEAR/3 (defic* OR insuffic* OR failure)):ti,ab,kw

#5 (TSH NEAR/2 Deficien*):ti,ab,kw

#6 (thyroid* low product*):ti,ab,kw

#7 (thyroid* under product*):ti,ab,kw

#8 (thyroid* underactive*):ti,ab,kw

#9 {OR #1-#8}

#10 MeSH descriptor: [Thyroxine] explode all trees

#11 (levothyrox* OR laevothyrox* OR levo-thyrox* OR laevo-thyrox* OR l-thyrox* OR levo-t OR L-T4 OR LT4):ti,ab,kw

#12 (berlthyrox OR dexnon OR eferox OR eltroxin OR eltroxine OR euthyrox OR eutirox OR "l thyrox" OR "l thyroxin henning" OR "l thyroxin beta" OR tetraiodothyronine OR "levo t" OR levothroid OR levothyroid OR levoxine OR levoxyl OR novothyral OR novothyrox OR oroxine OR q51bo43mg4 OR synthroid OR synthrox OR "t4 thyroid hormone" OR thevier OR thyrax OR "thyroid hormone, t4" OR thyroxin OR thyroxine OR tiroidine OR "tiroxina leo" OR unithroid):ti,ab,kw

#13 (Thyroid substitution therapy):ti,ab,kw

#14 {OR #10-#13}

#15 #9 AND #14 in Trials

***Database*: Ovid MEDLINE(R) ALL <since 1946 to January 26, 2024>**
**Search strategy:**

**1** exp Hypothyroidism/

**2** hypothyr*.mp.

**3** hypo-thyr*.mp.

**4** (thyroid? adj3 (defic* or insuffic* or failure?)).mp.

**5** (thyroid? adj3 low adj3 product*).mp.

**6** (thyroid? adj3 under adj3 product*).mp.

**7** (thyroid? adj3 underactive*).mp.

**8** (tsh adj3 defic*).mp.

**9** 1 or 2 or 3 or 4 or 5 or 6 or 7 or 8

**10** Thyroxine/

**11** (levothyrox* or laevothyrox* or levo-thyrox* or laevo-thyrox* or l-thyrox* or levo-t or L-T4 or LT4).mp.

**12** (3,5,3',5'-tetraiodothyronine or 51-48-9 or "7488-70-2 (thyroxine)" or berlthyrox or dexnon or eferox or eltroxin or eltroxine or euthyrox or eutirox or l thyrox or l thyroxin henning or l thyroxin beta or l thyroxine or l thyroxine roche or l-3,5,3',5'-tetraiodothyronine or levo t or levothroid or levothyroid or levoxine or levoxyl or novothyral or novothyrox or "o-(4-hydroxy-3,5-diiodophenyl) 3,5-diiodo-l-tyrosine" or "o-(4-hydroxy-3,5-diiodophenyl)-3,5-diiodotyrosine" or oroxine or q51bo43mg4 or synthroid or synthrox or t4 thyroid hormone or thevier or thyrax or thyroid hormone, t4 or thyroxin or thyroxine or tiroidine or tiroxina leo or unithroid).ti,ab.

**13** thyroid substitution therapy.mp.

**14** 10 or 11 or 12 or 13

**15** 9 and 14

**16** randomized controlled trial.pt.

**17** controlled clinical trial.pt.

**18** random*.ab.

**19** placebo.ab.

**20** clinical trials as topic.sh.

**21** random allocation.sh.

**22** trial.ti.

**23** 16 or 17 or 18 or 19 or 20 or 21 or 22

**24** exp animals/ not humans.sh.

**25** 23 not 24

**26** 15 and 25

***Database*: Embase <since 1974 to January 26, 2024>**

**Search strategy**

**1** exp Hypothyroidism/

**2** hypothyr*.mp.

**3** hypo-thyr*.mp.

**4** (thyroid? adj3 (defic* or insuffic* or failure?)).mp.

**5** (thyroid? adj3 low adj3 product*).mp.

**6** (thyroid? adj3 under adj3 product*).mp.

**7** (thyroid? adj3 underactive*).mp.

**8** (tsh adj3 defic*).mp.

**9** 1 or 2 or 3 or 4 or 5 or 6 or 7 or 8

**10** levothyroxine/

**11** (levothyrox* or laevothyrox* or levo-thyrox* or laevo-thyrox* or l-thyrox* or levo-t or L-T4 or LT4).mp.

**12** ("3,5,3',5'-tetraiodothyronine" or "51-48-9" or "7488-70-2 (thyroxine)" or berlthyrox or dexnon or eferox or eltroxin or eltroxine or euthyrox or eutirox or l thyrox or l thyroxin henning or l thyroxin beta or l thyroxine or l thyroxine roche or "l-3,5,3',5'-tetraiodothyronine" or levo t or levothroid or levothyroid or levoxine or levoxyl or novothyral or novothyrox or "o-(4-hydroxy-3,5-diiodophenyl) 3,5-diiodo-l-tyrosine" or "o-(4-hydroxy-3,5-diiodophenyl)-3,5-diiodotyrosine" or oroxine or q51bo43mg4 or synthroid or synthrox or t4 thyroid hormone or thevier or thyrax or "thyroid hormone, t4" or thyroxin or thyroxine or tiroidine or tiroxina leo or unithroid).ti,ab.

**13** thyroid substitution therapy.mp.

**14** 10 or 11 or 12 or 13

**15** 9 and 14

**16** Clinical-Trial/ or Randomized-Controlled-Trial/ or Randomization/ or Single-Blind-Procedure/ or Double-Blind-Procedure/ or Crossover-Procedure/ or Prospective-Study/ or Placebo/

**17** (((clinical or control or controlled) adj (study or trial)) or ((single or double or triple) adj (blind$3 or mask$3)) or (random$ adj (assign$ or allocat$ or group or grouped or patients or study or trial or distribut$)) or (crossover adj (design or study or trial)) or placebo or placebos).ti,ab.

**18** 16 or 17

**19** Animal/ not Human/

**20** 18 not 19

**21** 15 and 20

***Database*: APA PsycInfo <since 1806 until the 4^th^ week of January 2024>**
**Search strategy**

**1** exp Hypothyroidism/

**2** hypothyr*.tw.

**3** hypo-thyr*.tw.

**4** (thyroid? adj3 (defic* or insuffic* or failure?)).tw.

**5** (thyroid? adj3 low adj3 product*).tw.

**6** (thyroid? adj3 under adj3 product*).tw.

**7** (thyroid? adj3 underactive*).tw.

**8** (tsh adj3 defic*).tw.

**9** 1 or 2 or 3 or 4 or 5 or 6 or 7 or 8

**10** thyroxine/

**11** (levothyrox* or laevothyrox* or levo-thyrox* or laevo-thyrox* or l-thyrox* or levo-t or L-T4 or LT4).tw.

**12** (3,5,3',5'-tetraiodothyronine or 51-48-9 or "7488-70-2 (thyroxine)" or berlthyrox or dexnon or eferox or eltroxin or eltroxine or euthyrox or eutirox or l thyrox or l thyroxin henning or l thyroxin beta or l thyroxine or l thyroxine roche or l-3,5,3',5'-tetraiodothyronine or levo t or levothroid or levothyroid or levoxine or levoxyl or novothyral or novothyrox or "o-(4-hydroxy-3,5-diiodophenyl) 3,5-diiodo-l-tyrosine" or "o-(4-hydroxy-3,5-diiodophenyl)-3,5-diiodotyrosine" or oroxine or q51bo43mg4 or synthroid or synthrox or t4 thyroid hormone or thevier or thyrax or thyroid hormone, t4 or thyroxin or thyroxine or tiroidine or tiroxina leo or unithroid).tw.

**13** thyroid substitution therapy.tw.

**14** 10 or 11 or 12 or 13

**15** 9 and 14

***Web of Science***

**Search strategy**

1: TS=(hypothyr*) OR TS=(hypo-thyr*) OR TS=(thyroid$ NEAR/3 (defic* OR insuffic* OR failure$)) OR TS=(thyroid$ NEAR/3 low NEAR/3 product*) OR TS=(thyroid$ NEAR/3 under NEAR/3 product*) OR TS=(thyroid$ NEAR/3 underactive*) OR TS=(tsh NEAR/3 defic*)

2: TS=(levothyrox* OR laevothyrox* OR levo-thyrox* OR laevo-thyrox* OR l-thyrox* OR levo-t OR L-T4 OR LT4)

3: TS= clinical trial* OR TS=research design OR TS=comparative stud* OR TS=evaluation stud* OR TS=controlled trial* OR TS=follow-up stud* OR TS=prospective stud* OR TS=random* OR TS=placebo* OR TS=(single blind*) OR TS=(double blind*)

4: #3 AND #2 AND #1

**Search for observational studies until January 22, 2024**

***Database*: Ovid MEDLINE(R) ALL <dal 1946 al 22 gennaio 2024>**

**Search strategy**

**1** exp Hypothyroidism/

**2** hypothyr*.mp.

**3** hypo-thyr*.mp.

**4** (thyroid? adj3 (defic* or insuffic* or failure?)).mp.

**5** (thyroid? adj3 low adj3 product*).mp.

**6** (thyroid? adj3 under adj3 product*).mp.

**7** (thyroid? adj3 underactive*).mp.

**8** (tsh adj3 defic*).mp.

**9** 1 or 2 or 3 or 4 or 5 or 6 or 7 or 8

**10** Thyroxine/

**11** (levothyrox* or laevothyrox* or levo-thyrox* or laevo-thyrox* or l-thyrox* or levo-t or L-T4 or LT4).mp.

**12** (3,5,3',5'-tetraiodothyronine or 51-48-9 or "7488-70-2 (thyroxine)" or berlthyrox or dexnon or eferox or eltroxin or eltroxine or euthyrox or eutirox or l thyrox or l thyroxin henning or l thyroxin beta or l thyroxine or l thyroxine roche or l-3,5,3',5'-tetraiodothyronine or levo t or levothroid or levothyroid or levoxine or levoxyl or novothyral or novothyrox or "o-(4-hydroxy-3,5-diiodophenyl) 3,5-diiodo-l-tyrosine" or "o-(4-hydroxy-3,5-diiodophenyl)-3,5-diiodotyrosine" or oroxine or q51bo43mg4 or synthroid or synthrox or t4 thyroid hormone or thevier or thyrax or thyroid hormone, t4 or thyroxin or thyroxine or tiroidine or tiroxina leo or unithroid).ti,ab.

**13** 10 or 11 or 12

**14** Triiodothyronine/

**15** (triiodothyronine or liothyronine or T3 or LT3 or LT-3 or DTE or desiccated thyroid or thyroid extract).ti,ab.

**16** 14 or 15

**17** 13 and 16

**18** 9 and 17

**19** Observational Studies as Topic/

**20** Observational Study/

**21** exp Case-Control Studies/

**22** exp Cohort Studies/

**23** Controlled Before-After Studies/

**24** Historically Controlled Study/

**25**"comparative study".pt. or (comparative or "compared to" or comparing or comparison).ti,ab,kf. (6864937)

**26** case control$.tw.

**27** (cohort adj (study or studies)).tw.

**28** cohort analy$.tw.

**29** (follow up adj (study or studies)).tw.

**30** (observational adj (study or studies)).tw.

**31** longitudinal.tw.

**32** prospective.tw.

**33** retrospective.tw.

**34** 19 or 20 or 21 or 22 or 23 or 24 or 25 or 26 or 27 or 28 or 29 or 30 or 31 or 32 or 33 (9080685)

**35** 18 and 34

**36** exp "Animals"/ not "Humans"/

**37** 35 not 36

**Database: Embase <since 1974 to January 22, 2024>**

**Search strategy**

**1** exp Hypothyroidism/

**2** hypothyr*.mp.

**3** hypo-thyr*.mp.

**4** (thyroid? adj3 (defic* or insuffic* or failure?)).mp.

**5** (thyroid? adj3 low adj3 product*).mp.

**6** (thyroid? adj3 under adj3 product*).mp.

**7** (thyroid? adj3 underactive*).mp.

**8** (tsh adj3 defic*).mp.

**9** 1 or 2 or 3 or 4 or 5 or 6 or 7 or 8

**10** levothyroxine/

**11** (levothyrox* or laevothyrox* or levo-thyrox* or laevo-thyrox* or l-thyrox* or levo-t or L-T4 or LT4).mp.

**12** ("3,5,3',5'-tetraiodothyronine" or "51-48-9" or "7488-70-2 (thyroxine)" or berlthyrox or dexnon or eferox or eltroxin or eltroxine or euthyrox or eutirox or l thyrox or l thyroxin henning or l thyroxin beta or l thyroxine or l thyroxine roche or "l-3,5,3',5'-tetraiodothyronine" or levo t or levothroid or levothyroid or levoxine or levoxyl or novothyral or novothyrox or "o-(4-hydroxy-3,5-diiodophenyl) 3,5-diiodo-l-tyrosine" or "o-(4-hydroxy-3,5-diiodophenyl)-3,5-diiodotyrosine" or oroxine or q51bo43mg4 or synthroid or synthrox or t4 thyroid hormone or thevier or thyrax or "thyroid hormone, t4" or thyroxin or thyroxine or tiroidine or tiroxina leo or unithroid).ti,ab.

**13** 10 or 11 or 12

**14** liothyronine/

**15** (triiodothyronine or liothyronine or T3 or LT3 or LT-3 or DTE or desiccated thyroid or thyroid extract).ti,ab.

**16** 14 or 15

**17** 13 and 16

**18** 9 and 17

**19** Observational Study/

**20** exp case control study/

**21** exp cohort analysis/

**22** Controlled Before-After.mp.

**23** Historically Controlled Study.mp.

**24** comparative study/

**25** case control$.tw.

**26** case series.tw.

**27** (cohort adj (study or studies)).tw.

**28** cohort analy$.tw.

**29** (follow up adj (study or studies)).tw.

**30** (observational adj (study or studies)).tw.

**31** longitudinal.tw.

**32** prospective.tw.

**33** retrospective.tw.

**34** (comparative or "compared to" or comparing or comparison).ti,ab.

**35** or/19-34

**36** 18 and 35

**37** animal/ not human/

**38** 36 not 37

**VALUES, EQUITY, ACCEPTABILITY, FEASIBILITY**

**Database: Ovid MEDLINE(R) ALL <since 1946 to January 12, 2024>**

**Search strategy:**

**1** exp Hypothyroidism/

**2** hypothyr*.mp.

**3** hypo-thyr*.mp.

**4** (thyroid? adj3 (defic* or insuffic* or failure?)).mp.

**5** (thyroid? adj3 low adj3 product*).mp.

**6** (thyroid? adj3 under adj3 product*).mp.

**7** (thyroid? adj3 underactive*).mp.

**8** (tsh adj3 defic*).mp.

**9** 1 or 2 or 3 or 4 or 5 or 6 or 7 or 8

**10** Thyroxine/

**11** (levothyrox* or laevothyrox* or levo-thyrox* or laevo-thyrox* or l-thyrox* or levo-t or L-T4 or LT4).mp.

**12** (3,5,3',5'-tetraiodothyronine or 51-48-9 or "7488-70-2 (thyroxine)" or berlthyrox or dexnon or eferox or eltroxin or eltroxine or euthyrox or eutirox or l thyrox or l thyroxin henning or l thyroxin beta or l thyroxine or l thyroxine roche or l-3,5,3',5'-tetraiodothyronine or levo t or levothroid or levothyroid or levoxine or levoxyl or novothyral or novothyrox or "o-(4-hydroxy-3,5-diiodophenyl) 3,5-diiodo-l-tyrosine" or "o-(4-hydroxy-3,5-diiodophenyl)-3,5-diiodotyrosine" or oroxine or q51bo43mg4 or synthroid or synthrox or t4 thyroid hormone or thevier or thyrax or thyroid hormone, t4 or thyroxin or thyroxine or tiroidine or tiroxina leo or unithroid).mp.

**13** thyroid substitution therapy.mp.

**14** 10 or 11 or 12 or 13

**15** 9 and 14

**16** (acceptability or feasibility or adherence or compliance or satisfaction or utilization).mp.

**17** *Attitude to Health/

**18** *Patient Participation/

**19** *Patient Preference/

**20** (choice or choices).ti.

**21** value*.ti.

**22** health state values.ti,ab.

**23** valuation*.ti.

**24** expectation*.ti.

**25** attitude*.ti.

**26** acceptab*.ti,ab.

**27** point of view.ti,ab.

**28** user* participation.ti,ab.

**29** user* perspective*.ti,ab.

**30** patient* perce*.ti,ab.

**31** user* perce*.ti,ab.

**32** user view*.ti,ab.

**33** qualitative research/

**34** exp interviews as topic/

**35** questionnaires/

**36** narration/

**37** health care surveys/

**38** (qualitative$ or interview$ or focus group$ or questionnaire$ or survey$).tw.

**39** "critical interpretive synthes*".tw.

**40** (realist adj (review* or synthes*)).tw.

**41** (meta adj (method or triangulation)).tw.

**42** (CERQUAL or CONQUAL).tw.

**43** ((thematic or framework) adj synthes*).tw.

**44** ((patient$ or parent$ or famil$ or relative$ or carer$ or caregiver$ or care-giver$ or inpatient$ or in-patient$ or spous$ or husband$ or wife$ or wive$ or partner$ or mother$ or father$ or sibling$ or sister$ or brother$) adj6 (experience$ or belief$ or stress$ or emotion$ or anx$ or fear$ or concern$ or uncertain$ or unsure or thought$ or feeling$ or felt$ or view$ or opinion$ or perception$ or perspective$ or attitud$ or satisfact$ or know$ or understand$ or aware$)).ti,ab.

**45** exp consumer satisfaction/

**46** or/16-45

**47** 15 and 46

**48** (equit* or inequit* or inequalit* or disparit* or equality).tw.

**49** (ethnic* or race or racial* or racis*).tw.

**50** ((social* or socio-economic or socioeconomic or economic or structural or material) adj3 (advantage* or disadvantage* or exclude* or exclusion or include* or inclusion or status or position or gradient* or hierarch* or class* or determinant*)).tw.

**51** (health adj3 (gap* or gradient* or hierarch*)).tw.

**52** Vulnerable populations/ or socioeconomic factors/ or poverty/ or social class/ or Healthcare Disparities/ or Health Status Disparities/ or Poverty areas/ or Urban population/

**53** (SES or SEP or sociodemographic* or socio-demographic* or income or wealth* or poverty or educational level or level of education or educational attainment or well educated or better educated or unemploy* or home owner* or tenure or affluen* or well off or better off or worse off).tw.

**54** 48 or 49 or 50 or 51 or 52 or 53

**55** 15 and 54

**56** 47 or 55

**Database: Embase <since 1974 to January 12, 2024>**

**Search strategy:**

**1** exp *Hypothyroidism/

**2** hypothyr*.ti,ab.

**3** hypo-thyr*.ti,ab.

**4** (thyroid? adj3 (defic* or insuffic* or failure?)).ti,ab.

**5** (thyroid? adj3 low adj3 product*).ti,ab.

**6** (thyroid? adj3 under adj3 product*).ti,ab.

**7** (thyroid? adj3 underactive*).ti,ab.

**8** (tsh adj3 defic*).ti,ab.

**9** 1 or 2 or 3 or 4 or 5 or 6 or 7 or 8

**10** *levothyroxine/

**11** (levothyrox* or laevothyrox* or levo-thyrox* or laevo-thyrox* or l-thyrox* or levo-t or L-T4 or LT4).ti,ab.

**12** ("3,5,3',5'-tetraiodothyronine" or "51-48-9" or "7488-70-2 (thyroxine)" or berlthyrox or dexnon or eferox or eltroxin or eltroxine or euthyrox or eutirox or l thyrox or l thyroxin henning or l thyroxin beta or l thyroxine or l thyroxine roche or "l-3,5,3',5'-tetraiodothyronine" or levo t or levothroid or levothyroid or levoxine or levoxyl or novothyral or novothyrox or "o-(4-hydroxy-3,5-diiodophenyl) 3,5-diiodo-l-tyrosine" or "o-(4-hydroxy-3,5-diiodophenyl)-3,5-diiodotyrosine" or oroxine or q51bo43mg4 or synthroid or synthrox or t4 thyroid hormone or thevier or thyrax or "thyroid hormone, t4" or thyroxin or thyroxine or tiroidine or tiroxina leo or unithroid).ti,ab.

**13** thyroid substitution therapy.ti,ab.

**14** 10 or 11 or 12 or 13

**15** 9 and 14

**16** (acceptability or feasibility or adherence or compliance or satisfaction or utilization).mp.

**17** *attitude to health/

**18** *patient participation/

**19** *patient preference/

**20** (choice or choices).ti.

**21** value*.ti.

**22** health state values.ti,ab.

**23** valuation*.ti.

**24** expectation*.ti.

**25** attitude*.ti,ab.

**26** acceptab*.ti,ab.

**27** point of view.ti,ab.

**28** user* participation.ti,ab.

**29** user* perspective*.ti,ab.

**30** patient* perce*.ti,ab.

**31** user* perce*.ti,ab.

**32** user view*.ti,ab.

**33** qualitative research/

**34** exp interview/

**35** questionnaire/

**36** health care survey/

**37** (qualitative$ or interview$ or focus group$ or questionnaire$ or narrative$ or narration$ or survey$).tw.

**38** (CERQUAL or CONQUAL).tw.

**39** ((thematic or framework) adj synthes*).tw.

**40** ((patient$ or parent$ or famil$ or relative$ or carer$ or caregiver$ or care-giver$ or inpatient$ or in-patient$ or spous$ or husband$ or wife$ or wive$ or partner$ or mother$ or father$ or sibling$ or sister$ or brother$) adj6 (experience$ or belief$ or stress$ or emotion$ or anx$ or fear$ or concern$ or uncertain$ or unsure or thought$ or feeling$ or felt$ or view$ or opinion$ or perception$ or perspective$ or attitud$ or satisfact$ or know$ or understand$ or aware$)).ti.

**41** exp customer satisfaction/

**42** or/16-41

**43** 15 and 42

***Database*: APA PsycInfo <since 1806 until the 2^nd^ week of January 2024>**

**Search strategy:**

**1** exp Hypothyroidism/

**2** hypothyr*.tw.

**3** hypo-thyr*.tw.

**4** (thyroid? adj3 (defic* or insuffic* or failure?)).tw.

**5** (thyroid? adj3 low adj3 product*).tw.

**6** (thyroid? adj3 under adj3 product*).tw.

**7** (thyroid? adj3 underactive*).tw.

**8** (tsh adj3 defic*).tw.

**9** 1 or 2 or 3 or 4 or 5 or 6 or 7 or 8

**10** thyroxine/

**11** (levothyrox* or laevothyrox* or levo-thyrox* or laevo-thyrox* or l-thyrox* or levo-t or L-T4 or LT4).tw.

**12** (3,5,3',5'-tetraiodothyronine or 51-48-9 or "7488-70-2 (thyroxine)" or berlthyrox or dexnon or eferox or eltroxin or eltroxine or euthyrox or eutirox or l thyrox or l thyroxin henning or l thyroxin beta or l thyroxine or l thyroxine roche or l-3,5,3',5'-tetraiodothyronine or levo t or levothroid or levothyroid or levoxine or levoxyl or novothyral or novothyrox or "o-(4-hydroxy-3,5-diiodophenyl) 3,5-diiodo-l-tyrosine" or "o-(4-hydroxy-3,5-diiodophenyl)-3,5-diiodotyrosine" or oroxine or q51bo43mg4 or synthroid or synthrox or t4 thyroid hormone or thevier or thyrax or thyroid hormone, t4 or thyroxin or thyroxine or tiroidine or tiroxina leo or unithroid).tw.

**13** thyroid substitution therapy.tw.

**14** 10 or 11 or 12 or 13

**15** 9 and 14

**Appendix 4**

**Study selection for efficacy and safety**

**Systematic reviews**

**Study identification through databases**

Record excluded before screening:

Duplicates (n = 581)

Records identified from database search (n = 1479)

**Identificationne**

Record excluded for title and abstract (n = 895)

Records selected for title and abstract (n = 898)

Reports not found (n = 1)

Searched reports (n = 3)

**Screening**

Full text reports evaluated for elegibility (n = 2)

Systematic reviews excluded with reasons (n = 0)

Reviews included but not considered (n = 2)

**Inclusion**

**Randomized controlled trials**

**Study identification through databases**

Record excluded before screening:

Duplicates (n = 2832)

Records identified from database search (n = 7029)

**Identificationne**

Record excluded for title and abstract (n = 4172)

Records selected for title and abstract (n = 4197)

Searched reports (n = 25)

Reports not found (n = 0)

**Screening**

Reports excluded with reasons:

- Population not included (n = 1)
- Intervention (n = 1)
- Outcomes (n = 2)
- Type of study (n =5)

Full text reports evaluated for elegibility (n = 25)

Included studies:

- Comparison LT4 vs. LT4 + LT3 (n = 10)
- Comparison LT4 vs. LT3 (n = 2 in 4 papers)
- Comparison LT4 vs. DTE (n = 2)

**Inclusion**

**Observational studies**

**Study identification through databases**

Record excluded before screening:

Duplicates (n = 1481)

Records identified from database search (n = 4372)

**Identificationne**

Record excluded for title and abstract (n = 2876)

Records selected for title and abstract (n = 2891)

Searched reports (n = 11)

Reports not found (n = 0)

**Screening**

Reports excluded with reasons:

- Population not included (n = 4)
- Type of comparison (n = 4)
- Type of study (n = 3)

Full text reports evaluated for elegibility (n = 11)

Included studies(n = 0)

**Inclusion**

**Appendix 5**

**Evaluation of methodologic quality of included studies (checklist AMSTAR 2)**

| **Study** | **Item** | | | | | | | | | | | | | | | | **Global evaluation** |
| --- | --- | --- | --- | --- | --- | --- | --- | --- | --- | --- | --- | --- | --- | --- | --- | --- | --- |
|  | **1** | **2** | **3** | **4** | **5** | **6** | **7** | **8** | **9** | **10** | **11** | **12** | **13** | **14** | **15** | **16** |  |
| Millan-Alanis 2021 | Yes | Yes | No | Yes | Yes | Yes | No | Yes | Yes | No | No | Yes | Yes | Yes | No | Yes | Low |
| Lan et al, 2022 | Yes | Yes | No | Yes | Yes | No | No | Yes | Yes | No | Yes | No | No | No | Yes | Yes | Low |

# AMSTAR checklist.

1. Did the research questions and inclusion criteria for the review include the components of PICO*?*
2. Did the report of the review contain an explicit statement that the review methods were established prior to the conduct of the review and did the report justify any significant deviations from the protocol*?*
3. Did the review authors explain their selection of the study designs for inclusion in the review*?*
4. Did the review authors use a comprehensive literature search strategy*?*
5. Did the review authors perform study selection in duplicate*?*
6. Did the review authors perform data extraction in duplicate*?*
7. Did the review authors provide a list of excluded studies and justify the exclusions*?*
8. Did the review authors describe the included studies in adequate detail*?*
9. Did the review authors use a satisfactory technique for assessing the risk of bias in individual studies that were included in the review*?*
10. Did the review authors report on the sources of funding for the studies included in the review*?*
11. If meta-analysis (MA) was performed, did the review authors use appropriate methods for statistical combination of results*?*
12. If meta-analysis was performed, did the review authors assess the potential impact of risk of bias in individual studies on the results of the meta-analysis or other evidence synthesis*?*
13. Did the review authors account for risk of bias in primary studies when interpreting/discussing the results of the review*?*
14. Did the review authors provide a satisfactory explanation for, and discussion of, any heterogeneity observed in the results of the review*?*
15. If they performed quantitative synthesis did the review authors carry out an adequate investigation of publication bias (small study bias) and discuss its likely impact on the results of the review*?*
16. Did the review authors report any potential sources of conflict of interest, including any funding they received for conducting the review*?*

Appendix 6

Characteristics of the included RCTs

| **Comparison LT4 vs. LT4+LT3** | | | | | | |
| --- | --- | --- | --- | --- | --- | --- |
| **Study and year of publication (Country)** | **Study design** | **N** | **Characteristics of the** **participants** | **Interventions considered** | **Duration of treatment** | **Outcomes considered** |
| Appelholf et al, 2005 (Netherlands) | Parallel | 141 | On LT4 therapy for at least 6 months.  Cause of hypothyroidism: autoimmune hypothyroidism.  Women: 85.1%.  Mean age: 48.37 (9.54) years | 1. LT4/LT3 in a ratio of 10:1. 2. LT4/LT3 in a ratio of 5:1. 3. LT4. | 15 weeks | **Outcomes evaluated at baseline and after 5 and 15 weeks:** TSH, FT4, T3, SHBG, Anti-peroxidase Antibodies (TPO-Ab), lipid profile, osteocalcin.  Primary outcome: treatment preference.  Outcomes measured at 5, 10, and 15 weeks: psychological status measured with POMS; fatigue measured with the German version of the MFI-20; mental health measured with Rand-36 and SCL-90.  Outcomes measured at baseline and 15 weeks: neurocognitive outcomes (cognitive attention and memory) measured with the Wechsler Adult Intelligence Scale (WAIS), RBMT, CVLT, Digit Symbol subtest of the WAIS-III, Memory Comparison Task (MCT), and Paced Auditory Serial Attention Task.  Outcomes measured at baseline, 5, and 15 weeks: TSH, fT4, T3, SHBG, thyroid peroxidase antibodies (TPO-Ab), lipid profile, osteocalcin. |
| Biondi 2023 (Italy) | Parallel | 38 and 50 healthy controls | Patients after total thyroidectomy for differentiated thyroid cancer:   - At low risk during follow-up, according to ATA guidelines* - biochemical euthyroidism during LT4 monotherapy - undetectable thyroglobulin (Tg) levels - negative anti-thyroglobulin (TgAb) and anti-peroxidase (TPOAb) antibodies - negative neck ultrasound.   Women: 84.21%  Mean age: 46 (10.37) years | a) LT4+LT3.  b) LT4+placebo. | 12 months | Evaluation of biochemical and metabolic parameters of thyroid function (TSH, FT3, FT4, Tg, TgAb, TPOAb, cholesterol, triglycerides) and symptoms of hypothyroidism.  Cardiovascular outcomes: ECG and color-doppler echocardiography and changes in weight, waist circumference and hip circumference, systolic and diastolic blood pressure, heart rate.  Evaluation of compliance during combination therapy with LT4 + LT3.  Adverse events. |
| Brigante et al, 2024 (Italy) | Parallel | 141 | Patients diagnosed with hypothyroidism: age >18 years, without residual thyroid function (totally thyroidectomized for benign or malignant disease, with serum Tg <0.2 ng/mL and negative TgAb, to avoid interference in the Tg dosage method).  Women: 70.92%  Mean age: LT3+LT4, 55.5 (10.8) years; LT4, 56.4 (13) years. | - - 1. LT4+LT3 (n = 71).     2. LT4+placebo (n = 70). | 6 weeks, 3 months and 6 months | Primary outcome: serum SHBG changes after 6 months of treatment.  Secondary outcomes: BMI, changes in quality of life (assessed by ThyPRO questionnaire), TSH, FT3, FT4, total cholesterol, HDL-cholesterol, triglycerides, CTX, osteocalcin, and bone alkaline phosphatase. |
| Clyde et al, 2003 (USA) | Parallel | 46 | Patients receiving treatment for hypothyroidism for at least 6 months.  Cause of hypothyroidism: 70.55% autoimmune, 20.5% radioiodine treatment for toxic nodular goiter.  Women: 88.8%  Mean age: 44.15 (10.46) years. | LT4+LT3 *vs* LT4. | 16 weeks | Changes from baseline in:   - Neurocognitive outcomes, assessed with the attention and working memory test (WMS-III), paced auditory serial addition test, auditory consonant trigrams test, Thrustone Word fluency test, Trail-Making test. - Thyroid function: TSH, FT4, T3 levels; - depressive symptoms, measured with BDI; - quality of life, measured with the “Health-related of life questionnaire”. |
| Fadeyev et al, 2005 (Russia) | Parallel | 58 | Patients with autoimmune thyroiditis.  Women: 100%.  Mean age: 39.59 (9.14) years. | - Group A (n = 42): LT4 1.6 μg/kg. - Group B (n = 16): LT4 + LT3. - Group C (n = 20): healthy controls. | 24 weeks | Thyroid function: TSH, FT4, FT3 levels. Subgroup analysis based on TSH dosage: low-normal (<1.5 mU/L) and high-normal ≥1.5 mU/L). |
| Fadeyev et al, 2010 (Russia) | Parallel | 36 | Patients with untreated primary hypothyroidism.  Women: 100%.  Average age: group A, 43 years (34-46); group B, 40 years (32-44). | - Group A (n = 20): LT4 1.6 μg/kg. - Group B (n = 16): LT4 + LT3. | 24 weeks | Thyroid function: TSH, FT4, T3, T4, osteocalcin levels.  Lipid profile.  Echocardiographic parameters.  Symptoms of hypothyroidism measured with a 0-3 point-scale.  Preference for treatment. |
| Rodriguez et al, 2005 (USA) | Cross-over | 27 | Cause of hypothyroidism: autoimmune hypothyroidism, 77%; radioiodine treatment,13%; thyroidectomy, 10%.  Women: 83%.  Mean age: 47.5 (12.9) years. | LT4+LT3 vs. LT4. | 6 weeks | Primary outcome: fatigue measured by Piper Fatigue Scale.  Depressive symptoms measured by BDI-II and General Health Questionnaire-30 (GHQ-30).  Hypothyroidism symptoms, measured by VAS.  Cognitive outcomes (working memory), measured by Working Memory subtest of WMS-III.  Thyroid function: FT4, T4, T3.  Echocardiographic parameters.  Adverse events.  Subgroup analysis: low (n = 13) and high (n = 14) Piper Fatigue scale scores. |
| Saravanan et al, 2005 (UK) | Parallel | 697 | Patients with primary hypothyroidism, 71.6%.  Causes not reported.  Women: 83.78%.  Mean age: 57.34 (11.04) years. | LT4+LT3 (n = 344).  LT4 (n = 353). | 12 months | Outcomes assessed after 3 and 12 months.  Primary outcome: symptoms of hypothyroidism measured by GHQ-12, HADS and VAS.  Thyroid function: TSH, FT4, FT3, SHBG, TPOAb.  Satisfaction with treatment. |
| Sawka et al, 2007 (USA) | Parallel | 40 | Cause of hypothyroidism: NR.  Women: 90%.  Mean age: 47.25 (11.07) years | LT4+LT3 (n = 20).  LT4 (n = 20). | 15 weeks | Outcomes measured at baseline and after 2, 4, 6, 9, 12, and 15 weeks.  Depressive symptoms measured with SCL-90, Comprehensive Epidemiological Screens for Depression (CES-D).  Functional status/Quality of life, measured with MOS.  Thyroid function: TSH, FT4, FT3. |
| Valizadeh et al, 2009 (Iran) | Parallel | 71 | Cause of hypothyroidism: autoimmune, 76.65%; radioiodine, 20.05%; thyroidectomy, 3.3%.  Women: 80%.  Mean age: 39 years. | LT4+LT3 vs. LT4 | 16 weeks | Psychological well-being assessed with Goldberg’s general Health Questionnaire (GHQ-28).  Thyroid function: TSH, T4, T3.  Lipid profile.  Echocardiographic parameters. |
| **Comparison LT4 vs. LT3*** | | | | | | |
| Bjerkreim 2022a, Bjerkreim 2022b (NCT03627611) (Norway) | Cross-over | 69 | Patients with residual symptoms of hypothyroidism even if treated with LT4 (mean duration 10.6 ± 7.0 years).  Cause of hypothyroidism: autoimmune, 94.9%; post-surgery, 3.4%, radioiodine, 1.7%.  Women: 100%.  Mean age: 42.9 (9.7) years. | Phase 1 with LT3 or LT4 therapy for 12 weeks, then switch to the other treatment for another 12 weeks. | 12 weeks | Thyroid function: TSH, FT4, FT3, rT3.  Lipid profile, SHBG, other biomarkers, muscle strength.  Hypothyroidism symptoms and quality of life measured by ThyPRO, SF-36 and Fatigue Questionnaire.  Nonspecific and cardiovascular adverse events.  Treatment preferences. |
| Celi et al, 2010 (NCT00106119) (USA) | Cross-over | 10 | Patients aged ≥18 years thyroidectomized for multinodular goiter, adenoma, papillary carcinoma, medullary carcinoma, BMI 20-30 kg/m^2^ and replacement therapy with daily dose of LT4 ≥1.6 µg/kg.  Mean age: 51.3 (3.4) years. | LT4 vs. LT3 | 30 days | Thyroid function: TSH, T3, FT3, T4, FT4. |
| Celi et al, 2011 (NCT00106119) (USA) | Cross-over | 14 | Patients aged ≥18 years thyroidectomized for multinodular goiter, follicular adenoma, papillary carcinoma, follicular carcinoma, medullary carcinoma, Hashimoto’s thyroiditis, Graves’ disease, BMI 20-30 kg/m^2^ and replacement therapy with daily dose of LT4 ≥1.6 µg/kg.  Mean age: 49.3 (8) years. | LT4 vs. LT3 | 6 weeks | Physiological and anthropometric outcomes: weight, fat mass, systolic and diastolic blood pressure, heart rate, etc.  Laboratory outcomes: total cholesterol, LDL-cholesterol, HDL-cholesterol, triglycerides, etc.  Quality of life. |
| *For this comparison, cross-over RCTs were included in the narrative synthesis, due to the lack of parallel and cross-over RCTs reporting data to phase 1 prior to the switch. | | | | | | |
| **Comparison LT4 vs. DTE*** | | | | | | |
| Hoang et al, 2013 (USA) | Cross-over | 78 | Patients treated with LT4 for at least 6 months.  Cause of hypothyroidism: autoimmune, 50%; idiopathic, 20%; radioiodine, 14.29%; post-surgery, 11.43%; post-radioiodine, 4.28%.  Mean age: 46.22 (32.32) years. | DTE vs. LT4 | 16 weeks | Reduction of hypothyroid symptoms.  Quality of life measured by TSQ and GHQ-12.  Depressive symptoms measured by BDI.  Thyroid function: TSH, FT4, T4, SHBG levels.  Cognitive outcomes measured by WMS-IV.  Other outcomes: weight, lipid profile (total, LDL, and HDL cholesterol). |
| Shakir et al, 2021 (USA) | Cross-over | 75 | Cause of hypothyroidism: autoimmune, 61.3%; Graves’ disease, 5.3%, post-thyroidectomy, 21.3%; idiopathic, 12.1%.  Women: 77.3%.  Mean age: 50 (range 29-65) | LT4+LT3 vs.LT4 vs. DTE | 22 weeks | Reduction of hypothyroid symptoms.  Quality of life measured by TSQ and GHQ-12.  Depressive symptoms measured by BDI.  Thyroid function: TSH, FT4, T4, SHBG levels.  Other outcomes: weight, lipid profile (total cholesterol, LDL-cholesterol, HDL-cholesterol).  Adverse events.  Adherence to treatment. |
| * For this comparison, crossover RCTs were included in the narrative synthesis, due to the lack of parallel and crossover RCTs reporting data to phase 1 prior to switching*.*  Legend  BDI = Beck Depression Inventory. CVLT = California Verbal Learning Test. GHQ-12 = QoL general health questionnaire-12. GHQ-30 = General Health Questionnaire-30. MFI-20 = Multidimensional Fatigue Inventory. MOS = Medical Outcomes Study. Rand-36 = Rand 36-item health survey. RBMT = Rivermead Behavioral Memory Test. SF-36= Short form 36. TSQ = thyroid symptom questionnaire. VAS = visual analogue scale. WMS-IV = Wechsler memory scale-version IV. | | | | | | |
